# Novel therapeutic strategy for cervical cancer harboring *FGFR3-TACC3* fusions

**DOI:** 10.1038/s41389-017-0018-2

**Published:** 2018-01-23

**Authors:** Ryo Tamura, Kosuke Yoshihara, Tetsuya Saito, Ryosuke Ishimura, Juan Emmanuel Martínez-Ledesma, Hu Xin, Tatsuya Ishiguro, Yutaro Mori, Kaoru Yamawaki, Kazuaki Suda, Seiya Sato, Hiroaki Itamochi, Teiichi Motoyama, Yoichi Aoki, Shujiro Okuda, Cristine R. Casingal, Hirofumi Nakaoka, Ituro Inoue, Roel G. W. Verhaak, Masaaki Komatsu, Takayuki Enomoto

**Affiliations:** 1 Department of Obstetrics and Gynecology, Niigata University Graduate School of Medical and Dental Sciences, Niigata, Japan; 2 Department of Biochemistry, Niigata University Graduate School of Medical and Dental Sciences, Niigata, Japan; 3 Department of Bioinformatics and Computational Biology, The University of Texas MD Anderson Cancer Center, Houston, TX USA; 4 Department of Obstetrics and Gynecology, Tottori University School of Medicine, Tottori, Japan; 5 Department of Molecular and Diagnostic Pathology, Niigata University Graduate School of Medical and Dental Sciences, Niigata, Japan; 6 Department of Obstetrics and Gynecology, Graduate School of Medicine, University of the Ryukyus, Okinawa, Japan; 7 Department of Bioinformatics, Niigata University Graduate School of Medical and Dental Sciences, Niigata, Japan; 8 Division of Human Genetics, National Institute of Genetics, Mishima, Japan; 9 Jackson Laboratory for Genomic Medicine, Farmington, CT USA

## Abstract

We previously found that therapeutic targetable fusions are detected across various cancers. To identify therapeutic targetable fusion in uterine cervical cancer, for which no effective gene targeted therapy has yet been clinically applied, we analyzed RNA sequencing data from 306 cervical cancer samples. We detected 445 high confidence fusion transcripts and identified four samples that harbored *FGFR3-TACC3* fusion as an attractive therapeutic target. The frequency of *FGFR3-TACC3*-fusion-positive cervical cancer is also 1.9% (2/103) in an independent cohort. Continuous expression of the *FGFR3-TACC3* fusion transcript and protein induced anchorage-independent growth in the cervical epithelial cell line established from the ectocervix (Ect1/E6E7) but not in that from endocervix (End1/E6E7). Injection of *FGFR3-TACC3* fusion-transfected-Ect1/E6E7 cells subcutaneously into NOG mice generated squamous cell carcinoma xenograft tumors, suggesting the association between *FGFR3-TACC3* fusion and squamous cell carcinogenesis. Transfection of a *FGFR3-TACC3* fusion transcript into four cervical cancer cell lines (SiHa, ME180, HeLa, and Ca Ski) induced activation of the MAPK pathway and enhancement of cell proliferation. Transcriptome analysis of the *FGFR3-TACC3* fusion-transfected cell lines revealed that an *IL8*-triggered inflammatory response was increased, via activation of FGFR3–MAPK signaling. Continuous expression of *FGFR3-TACC3* fusion led to activation of the PI3K–AKT pathway only in the two cell lines that harbored *PIK3CA* mutations. Sensitivity to the *FGFR* inhibitor, BGJ398, was found to depend on *PIK3CA* mutation status. Dual inhibition of both FGFR and AKT showed an obvious synergistic effect in cell lines that harbor mutant *PIK3CA*. Additionally, TACC3 inhibitor, KHS101, suppressed FGFR3-TACC3 fusion protein expression and showed antitumor effect against *FGFR3-TACC3* fusion-transfected cell lines. *FGFR3-TACC3* fusion-positive cancer has frequent genetic alterations of the PI3K/AKT pathway and selection of appropriate treatment based on PI3K/AKT pathway status should be required.

## Introduction

Among women worldwide, uterine cervical cancer is the third most common cancer, the fourth most frequent cause of cancer-related death^1^, and is mainly caused by persistent infection with oncogenic strains of human papillomavirus (HPV)^2^. Although highly safe and effective prophylactic vaccines against the most oncogenic types of HPV are widely available, the rates for HPV vaccinations are lower than those for other routine childhood vaccinations^3,4^. In addition, the cervical cancer screening rate for women is relatively low and the incidence of invasive cervical cancer has not been decreasing. Although early stage cervical cancer is highly curable, advanced stage or metastatic cervical cancers are still difficult to treat; thus, the development of better therapeutic strategies is an urgent task.

To that end, the Cancer Genome Atlas (TCGA) Research Network recently performed an extensive molecular characterization of 228 invasive cervical cancers and detected several new therapeutic targets^5^. However, bringing these candidates into clinical settings will require further functional analysis. In addition, several pan-cancer studies have identified fusion genes as attractive therapeutic targets across many tumor types^6, 7^. Our pan-cancer fusion study^6^ showed that a number of cancers have targetable *FGFR3-TACC3* fusion. Therefore, we planned to discover and validate the presence of therapeutically targetable events such as *FGFR3-TACC3* fusion in cervical cancer using datasets from two large cervical cancer patient cohorts, and then to clarify the use of the fusion product as a therapeutic target, leading to the development of new therapeutic strategies for cervical cancer.

## Results

### Identification of *FGFR3-TACC3* fusion-positive cervical cancer

**Fig. 1:**
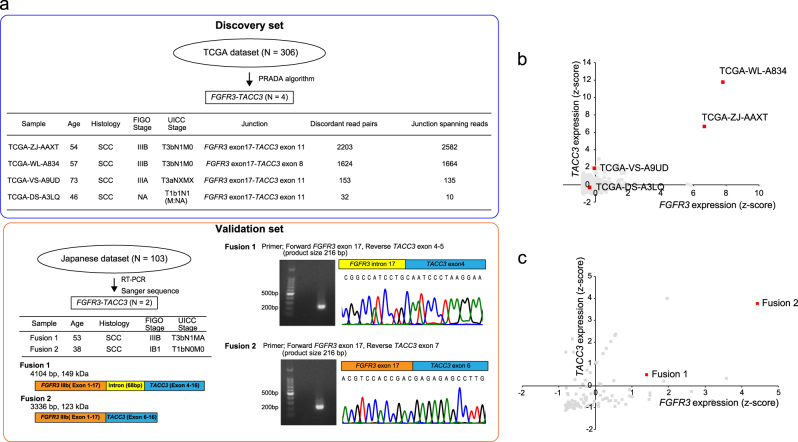
Identification of *FGFR3-TACC3* fusion in TCGA and the Japanese cervical cancer datasets. (**a**) Frequency of *FGFR3-TACC3* fusion-positive cervical cancer in TCGA and Japanese datasets. Clinical information of *FGFR3-TACC3* fusion-positive cervical cancer and two types of *FGFR3-TACC3* fusion transcripts detected in the Japanese dataset are shown. **b**, **c** Scatter plot of *FGFR3* and *TACC3* mRNA expression in (**b**) TCGA dataset (*n *= 306) or (**c**) Japanese dataset (*n* = 103). *FGFR3-TACC3* fusion-positive samples are highlighted in red

We downloaded TCGA RNA sequencing data obtained from 306 uterine cervical cancer samples from the Cancer Genome Hub (CGHub, https://cghub.ucsc.edu). We detected 445 high confidence fusion transcripts by using the Pipeline for RNA sequencing Data Analysis (PRADA)^8^. Of 306 TCGA cervical cancer samples, we identified four *FGFR3-TACC3* fusion-positive samples. We also found two additional *FGFR3-TACC3* fusion-positive cases in a Japanese cohort of 103 patients with cervical cancer by using RT-PCR and Sanger sequencing (Fig. 1a). The frequency of *FGFR3-TACC3* fusion-positive cervical cancer is similar in the two large cervical cancer patient cohorts (1.3% and 1.9%, respectively). We did not identify any other recurrent kinase fusions. All *FGFR3-TACC3* fusion-positive samples preserved the kinase domain of *FGFR3*, but showed a variety of gene expression patterns in both *FGFR3* and *TACC3* (Figs. 1a–c). All six *FGFR3-TACC3* fusion-positive samples were histologically diagnosed as squamous cell carcinoma, which corresponds well to the finding that *FGFR3-TACC3* fusions are detected in TCGA lung squamous cell carcinoma but not in TCGA lung adenocarcinoma (Fig. 1a and Supplementary Table 1).

### Significance of the *FGFR3-TACC3* fusion gene in cervical cancer

**Fig. 2:**
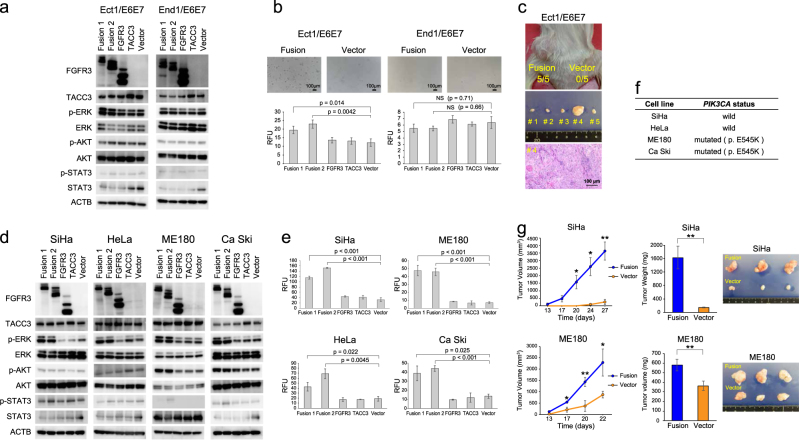
Frequency and molecular characteristics of *FGFR3-TACC3* fusion in cervical cancer. **a**, **d** Alteration of protein expression in the FGFR signaling pathway after transfection of Fusion 1, Fusion 2, wild-type FGFR3, wild-type TACC3, and control vector into **a** cervical epithelial or **d** cancer cell lines. **b**, **e** Cells were seeded at 1.0 × 10^3^ cells/well in a 96-well plate. Colony-forming ability was tested at day 14 in cervical epithelial cell lines, day 7 in SiHa, day 8 in HeLa, and ME180 and day 10 in Ca Ski cells. Bar graph represents average ± standard deviation of three independent experiments (unpaired *t*-test). Relative fluorescence unit (RFU) was used to assess colony-forming ability. Scale bar was 100 μm. **c**, **g** Subcutaneous injection of *FGFR3-TACC3* fusion-transfected Ect1/E6E7, SiHa, and ME180 cells led to occurrence of squamous cell carcinoma in NOG mouse. Five NOG mice were injected with 2.0 × 10^6^ cells (left: *FGFR3-TACC3* fusion, right: control vector), and sacrificed 105 days (Ect1/E6E7), 27 days (SiHa), and 22 days (ME180) after cell implantation. (**c**) Representation of hematoxylin and eosin staining for xenograft tumor derived from *FGFR3-TACC3* fusion-transfected Ect1/E6E7 (×400 magnification). **g** Volume and weight of xenograft tumors in fusion-transfected SiHa and ME180 cells. Line and bar graphs represent average ± standard deviation of three independent experiments (unpaired *t*-test; *p < 0.05; ** *p* < 0.01). **f** *PIK3CA* gene status of four cervical cancer cell lines used in this study

To understand the biological significance of the *FGFR3-TACC3* fusion event in cervical cancer^9,10^, we transfected two different gene-junction types of *FGFR3-TACC3* (designated “Fusion 1” and “Fusion 2”), which we had detected in the Japanese cohort, into two cervical epithelial cell lines established from the ectocervix (Ect1/E6E7) and endocervix (End1/E6E7) of the same patient. We found continuous expression of the *FGFR3-TACC3* fusion transcript and protein-induced increased phosphorylation of ERK (Fig. 2a) and anchorage-independent growth (Fig. 2b) in Ect1/E6E7, but not in End1/E6E7. By injecting *FGFR3-TACC3* fusion-transfected Ect1/E6E7 cells subcutaneously into NOG mice, squamous cell carcinoma xenograft tumors were generated (Fig. 2c and Supplementary Figure. 1a). No tumor was formed by injecting *FGFR3-TACC3* fusion-negative Ect1/E6E7 cells into NOG mice. *FGFR3-TACC3* fusion was therefore clearly associated with tumorigenesis of cervical squamous cell carcinoma.

Next, we transfected a *FGFR3-TACC3* fusion transcript into four cervical cancer cell lines (SiHa, ME180, HeLa, and Ca Ski). Continuous expression of the FGFR3-TACC3 fusion protein led to activation of the MAPK pathway via increased phosphorylation of ERK. A colony formation assay demonstrated that the number of colonies increased significantly in all cervical cancer cells transfected with *FGFR3-TACC3* fusion, compared to those transfected with a control vector (Figs. 2d, e). Increased phosphorylation of AKT was observed only in the two *FGFR3-TACC3* fusion-transfected cell lines that harbored a *PIK3CA*-activating mutation (ME180 and Ca Ski) (Figs. 2d, f). ME180 and Ca Ski cell lines had no other common genetic alterations leading to activation of the PI3K–AKT pathway (Supplementary Table 2), suggesting that activation of the AKT pathway in these two *FGFR3-TACC3* fusion-transfected cell lines might be associated with their mutated/activated *PIK3CA* status.

After subcutaneous injection of *FGFR3-TACC3* fusion-transfected SiHa and ME180 cells, rapid tumor growth was observed compared to that among the cells transfected with the control vector (Fig. 2g). The xenograft tumors derived from *FGFR3-TACC3* fusion-transfected SiHa and ME180 cells showed an obvious reduction of their keratinizing area, along with rapid tumor growth, indicating the possibility of conversion to a poorly differentiated tumor (Supplementary Figure 1b). There was overexpression of the *FGFR3-TACC3* fusion transcript and the kinase domain portion of the FGFR3 protein within the fusion protein in the xenograft tumors (Supplementary Figures 1c and d).

### Induction of inflammatory response via activated FGFR–MAPK signaling pathway

**Fig. 3:**
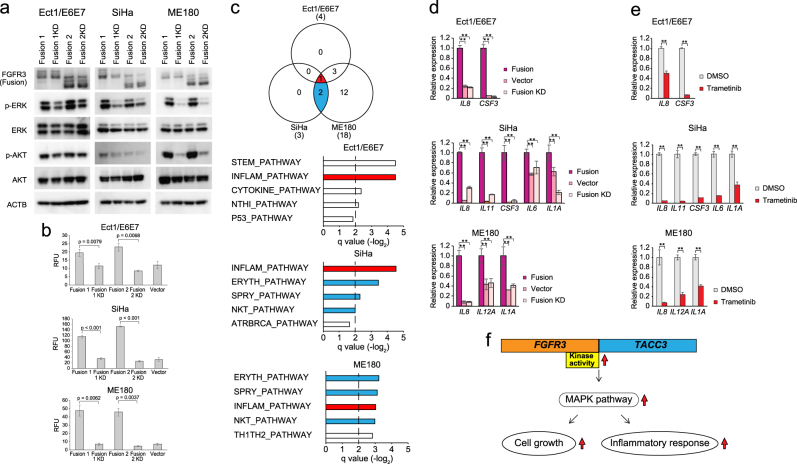
*FGFR3-TACC3* fusion induced not only cell proliferation but also inflammation responses via MAPK pathway. **a** Alteration of protein expression in the FGFR signaling pathway after transfection of Fusion 1, Fusion 1 KD, Fusion 2, and Fusion 2 KD into Ect1/E6E7, SiHa, and ME180 cell lines. **b** Comparison of colony-forming ability between *FGFR3-TACC3* fusion- vs. *FGFR3-TACC3* KD fusion-transfected cells. Cells were seeded at 1.0 × 10^3^ cells/well in a 96-well plate. Colony-forming ability was tested at day 14 in Ect1/E6E7, day 7 in SiHa, and day 8 in ME180 cells. **c** Venn diagram showing number of common overrepresented pathways among *FGFR3-TACC3* fusion-transfected Ect1/E6E7, SiHa, and ME180 cell lines. Top five ranked overrepresented pathways based on GSEA between *FGFR3-TACC3* fusion group and control group are shown per cell line. **d**, **e** Relative expression of representative genes in the inflammatory response-related gene sets among *FGFR3-TACC3* fusion, *FGFR3-TACC3* KD fusion, control groups (Ect1/E6E7, SiHa, and ME180) **d** before and **e** after treatment with trametinib for 24 h were evaluated using quantitative real-time RT-PCR. Bar graph represents average genes in overrepresented pathways based on GSEA (unpaired *t*-test; ** denotes *p* < 0.01). **f** Alterations of cell proliferation and inflammation responses via the MAPK pathway in *FGFR3-TACC3* fusion-positive cancer cells

We transfected a “kinase-dead mutant” *FGFR3-TACC3* fusion transcript (“*FGFR3-TACC3* KD fusion”) into Ect1/E6E7, SiHa, and ME180 to investigate the significance of *FGFR3* kinase activity in *FGFR3-TACC3* fusion-positive cervical cancer. Overexpression of *FGFR3*-*TACC3* KD fusion induced neither phosphorylation of downstream ERK nor phosphorylation of AKT in any of the four cell lines (Fig. 3a). The colony-forming ability of *FGFR3*-*TACC3* KD fusion-transfected cell lines was reduced, compared with that of the *FGFR3-TACC3* fusion-transfected cell lines (Fig. 3b). These findings confirm previous reports^9,11^ that *FGFR3* kinase activity plays an important role in the pathogenesis of *FGFR3-TACC3* fusion-positive cervical cancers.

In addition, we performed RNA sequencing for Ect1/E6E7, SiHa, and ME180 to compare gene expression profiles between the *FGFR3-TACC3* fusion, *FGFR3-TACC3* KD fusion, and control transfection groups. RNA sequencing data confirmed that *FGFR3-TACC3* fusion and *FGFR3-TACC3* KD fusion transcripts were correctly transfected and expressed in all four cell lines (Supplementary Table 3). Interestingly, gene set enrichment analysis (GSEA) demonstrated that an inflammatory response pathway was commonly activated in the *FGFR3-TACC3* fusion transfection group compared to the control group, and many overrepresented pathways were similar between *FGFR3-TACC3* fusion-transfected SiHa and ME180 cell lines, regardless of *PIK3CA* status (Fig. 3c and Supplementary Table 4).

After validating the expression of representative genes from the inflammatory pathway through quantitative RT-PCR (Fig. 3d), we investigated whether *FGFR3-TACC3* fusion induced inflammation via the MAPK pathway. By using an MEK inhibitor (trametinib) to inhibit MAPK signaling in the *FGFR3-TACC3* fusion-transfected cell lines, the expression levels of inflammatory response genes were significantly suppressed (Fig. 3e). Leading edge analysis suggested that *IL8*, which was associated with progression of cervical cancer^12,13^, might trigger inflammation in *FGFR3-TACC3* fusion-positive cervical cancer (Supplementary Figure 2). Accordingly, IL-8 secretion was higher in the *FGFR3-TACC3* fusion group compared to the other two groups, and suppression of the MAPK pathway via tramenitib reduced IL-8 secretion in the *FGFR3-TACC3* fusion group (Supplementary Figure 3). When we performed GSEA between the *FGFR3-TACC3* KD fusion group and the control group, there were few if any overrepresented pathways in the *FGFR3-TACC3* KD group (Supplementary Figure 4). Corresponding to a previous report that IL-8 is a transcription activation target of RAS signaling^14^, our transcriptome analysis revealed that *FGFR3* kinase-dependent activation of MAPK signaling would promote not only cell proliferation but also an inflammatory response^15^ in *FGFR3-TACC3* fusion-positive cervical cancer (Fig. 3f).

### Sensitivity to *FGFR* inhibitor (BGJ398) was dependent on PI3K/AKT status

**Fig. 4:**
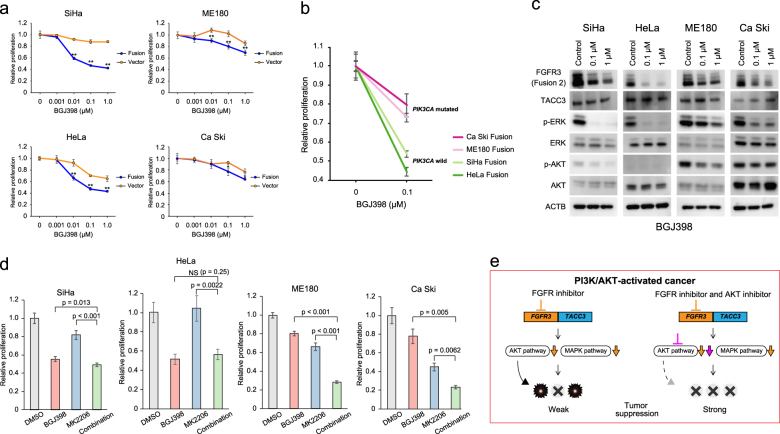
Sensitivity to *FGFR* inhibitor (BGJ398) was dependent on *PIK3CA* mutation status. **a** Cell proliferation was assessed by Cell Titer Glo assay after 72 h of treatment with different concentrations of BGJ398 in *FGFR3-TACC3* fusion-transfected cervical cancer cell lines (SiHa, HeLa, ME180, and Ca Ski). Bar graph represents average ± standard deviation of quadruplicate experiments (unpaired *t*-test; ** denotes *p* < 0.01). **b** Comparison of cell proliferation after 72 h of treatment with BGJ398 (0.1 μg/ml) between *FGFR3-TACC3* fusion-transfected cell lines with and without *PIK3CA* mutation. **c** Western blot analysis in *FGFR3-TACC3* fusion-transfected cell lines (SiHa, HeLa, ME180, and Ca Ski) after 24 h of treatment with different concentrations of FGFR inhibitor (BGJ398). **d** Cell proliferation after 72 h of treatment with FGFR inhibitor (BGJ398; 0.1 μg/ml), AKT inhibitor (MK2206; 0.5 μg/ml), or combination of BGJ398 and MK2206. NS denotes not significant. Bar graph represents average ± standard deviation of quadruplicate experiments. **e** Mechanism of enhanced efficacy of combined inhibition of FGFR and AKT in PI3K/AKT-activated *FGFR3-TACC3* fusion-positive cancer

Based on the molecular characteristics of *FGFR3-TACC3* fusion-transfected cervical cancer, we searched for effective molecular target inhibitors. First, we assessed the antitumor effect of the FGFR inhibitor BGJ398, which showed strong effectiveness against cells carrying FGFR aberrations in previous studies^9,16^. *FGFR3-TACC3* fusion-transfected cell lines were generally more sensitive to this FGFR inhibitor compared to controls (Fig. 4a). However, sensitivity to FGFR inhibition for *FGFR3-TACC3* fusion-transfected ME180 and Ca Ski cell lines that harbored *PIK3CA*-activating mutations was relatively lower than that for the fusion-transfected SiHa and HeLa cell lines that both carried a wild-type *PIK3CA* gene (Figs. 4a, b). Corresponding to the differences in sensitivity, western blot analysis after treatment with the FGFR inhibitor showed that expression of phosphorylated ERK and AKT remained high in ME180 and Ca Ski cells that carried mutant *PIK3CA* (Fig. 4c).

Next, we evaluated the effectiveness of the combination treatment. Although treatment with the MEK inhibitor trametinib alone induced decreases in phosphorylated ERK, leading to an antitumor effect (Supplementary Figures 5a and b), the combination treatment with the FGFR inhibitor and the MEK inhibitor showed an antitumor effect that was far less than expected, especially in *FGFR3-TACC3* fusion-transfected cells that carried a *PIK3CA* mutation (Supplementary Figure 6).

Meanwhile, there was no difference in sensitivity to the AKT inhibitor MK2206 between the *FGFR3-TACC3* fusion-transfected and control-transfected cell lines (Supplementary Figures 5c and d). Dual inhibition of both FGFR and AKT showed an obvious synergistic effect in the fusion-transfected ME180 and Ca Ski cell lines that harbored mutant *PIK3CA*, but little added effect in SiHa and HeLa cells that harbored wild-type *PIK3CA* (Fig. 4d). Previous studies reported that the PI3K/AKT pathway is activated in about 30% of cervical cancers^5,17^, and four of six *FGFR3-TACC3* fusion-positive cervical cancer samples showed activation of the PI3K–AKT pathway due to copy number amplification or a somatic mutation of at least one gene in the PI3K–AKT pathway (Supplementary Figure 7 and Supplementary Table 5). This would help explain why the combination therapy with FGFR3 and AKT inhibitors was so effective against PI3K–AKT-activated cervical cancer cells that harbored *FGFR3-TACC3* fusion (Fig. 4e).

### The effectiveness of TACC3 inhibitor, KHS101, for FGFR3-TACC3 fusion-positive cancer

**Fig. 5:**
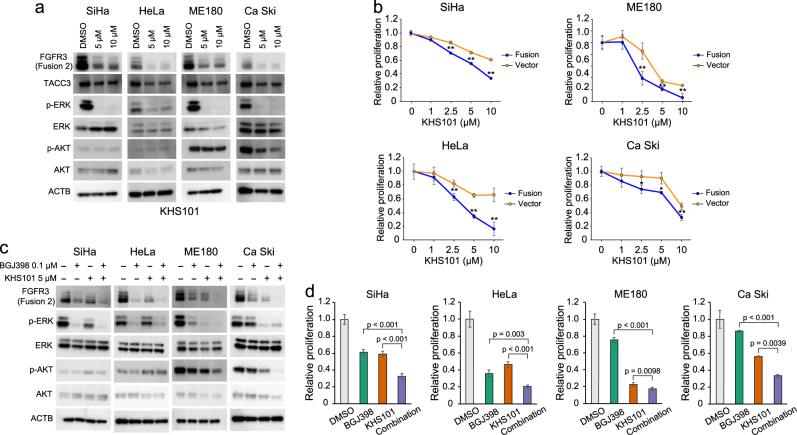
Effectiveness of combined inhibition of FGFR and TACC3 in *FGFR3-TACC3* fusion-positive cancer. **a** Alterations of protein expression in the FGFR signaling pathway in *FGFR3-TACC3* fusion-transfected cell lines (SiHa, HeLa, ME180, and Ca Ski) after 24 h of treatment with TACC3 inhibitor (KHS101). **b** Cell proliferation based on Cell Titer Glo assay after 72 h of treatment with BGJ398 (0.1 μg/ml), KHS101 (5.0 μg/ml), or the combination of BGJ398 and KHS101. Bar graph represents average ± standard deviation of quadruplicate experiments (unpaired *t*-test; ** denotes *p* < 0.01). **c** Alterations of protein expression in FGFR signaling pathway in *FGFR3-TACC3* fusion-transfected cell lines (SiHa, HeLa, ME180, and Ca Ski) after 24 h of treatment with control, BGJ398 (0.1 μg/ml), KHS101 (5.0 μg/ml), or the combination of BGJ398 and KHS101. **d** Cell proliferation based on Cell Titer Glo assay after 72 h of treatment with BGJ398 (0.1 μg/ml), KHS101 (5.0 μg/ml), or the combination of BGJ398 and KHS101. Mechanism of enhanced efficacy of combined inhibition of FGFR and TACC3 in PI3K/AKT neutral or activated *FGFR3-TACC3* fusion-positive cancer

We focused on the gene fusion partner *TACC3*^18,19^ as a possible therapeutic target. Surprisingly, the TACC3 inhibitor KHS101^20^ reduced not only wild-type TACC3 protein expression but also expression of the FGFR3-TACC3 fusion protein in *FGFR3-TACC3* fusion-transfected cervical cancer cell lines, leading to suppression of phosphorylation of ERK and AKT (Fig. 5a). The *FGFR3-TACC3* fusion-transfected cell line group was more sensitive to KHS101 compared to the control group (Fig. 5b). Compared to the FGFR inhibitor or TACC3 inhibitor alone, dual inhibition of FGFR and TACC3 demonstrated significant reduction of the FGFR3-TACC3 fusion protein and phosphorylation of ERK and AKT (Fig. 5c), leading to a synergistic suppression of cell proliferation in *FGFR3-TACC3* fusion-transfected cervical cancer cells (Fig. 5d).

## Discussion

In this study, we clarified the frequency and biological significance of the *FGFR3-TACC3* fusion gene in uterine cervical cancer by combining transcriptomic analysis of clinical samples and in vitro/in vivo experiments. We used the molecular characteristics of *FGFR3-TACC3* fusion-positive cervical cancer to develop a new therapeutic strategy for this disease.

Since it was discovered in 2012^9^, the *FGFR3-TACC3* fusion gene has been detected in glioblastoma multiforme^9,10^, bladder cancer^21^, lung cancer^22^, nasopharyngeal carcinoma^23^, and uterine cervical cancer^24,25^. In our previous fusion study that analyzed RNA sequencing data derived from 4366 primary tumor samples across 13 tumor types, we found the *FGFR3-TACC3* fusion gene to represent the most frequent kinase in-frame fusion and to be detected more in squamous cell carcinoma compared to adenocarcinoma^6^. In line with this previous result, we detected *FGFR3-TACC3* fusions only in uterine cervical squamous cell carcinoma but not adenocarcinoma (Fig. 1a and Supplementary Table 1) and demonstrated that transfection of *FGFR3-TACC3* fusion transformed the cervical epithelial cell line established from the ectocervix (Ect1/E6E7) to squamous cell carcinoma. Therefore, *FGFR3-TACC3* fusion might play an important role in the tumorigenesis of squamous cell carcinoma.

Cell lines that express *FGFR3-TACC3* fusion are sensitive to FGFR inhibition^26,27^, and *FGFR3-TACC3* fusion is a molecular characteristic that is susceptible to FGFR inhibitors. Indeed, several FGFR inhibitors have been developed and are being assessed in cancers that harbor oncogenic *FGFR* alterations, including *FGFR3-TACC3* fusion. Helsten et al.^25^ reported that 343 of 4853 cancer patients (7.1%) have at least one FGFR aberration and the percentage of cases with FGFR aberrations is less than 10% in most cancer types except urothelial, breast, and endometrial cancers. Therefore, to assess the effectiveness of FGFR inhibitors, it may be suitable to design a basket trial that enrolls patients who have various types of cancer but have the same genomic alteration. However, because FGFR aberrations do not always act in the same way across cancers, we need to consider the genomic background of the cancer when we select FGFR inhibitors as therapy for cancers with FGFR alterations.

For instance, PI3K–AKT signaling is a key pathway to use in determining the efficacy of FGFR inhibitors for cancers that harbor FGFR alterations. Our findings show that activation of PI3K–AKT signaling in *FGFR3-TACC3* fusion-transfected cell lines was linked to the status of *PIK3CA* and that *FGFR3-TACC3* fusion-transfected cell lines with activated PI3K–AKT signaling were refractory to the FGFR inhibitor, suggesting that *PIK3CA* mutation status might be a biomarker of FGFR inhibition. Corresponding to our results, Wang et al.^28^ clarified that the inhibition of PIK3CA acts synergistically with FGFR inhibitors in a *FGFR3-TACC3* fusion-positive cell line. Similarly, proteomic analysis between FGFR inhibitor-resistant and inhibitor-sensitive cell lines that harbor *FGFR3-TACC3* fusions demonstrates increased phosphorylation of Akt (T308 and S473) and its downstream target GSK3 (S9 and S21) in the inhibitor-resistant cell line compared to the inhibitor-sensitive cell line^27^. Genetic alterations in the PI3K–AKT signaling pathway were observed in about 35–67% of *FGFR3-TACC3* fusion-positive clinical samples (Supplementary Table S5).^25^ Therefore, we would need to examine PI3K/AKT status when we treat *FGFR3-TACC3* fusion-positive cancer with the FGFR inhibitor.

Although several kinase inhibitors are clinically utilized to treat oncogenic kinase fusion-positive cancer (e.g., ALK inhibitor to treat ALK fusion-positive lung cancer), at present^29^, acquired resistance to kinase inhibitors has become a problem in clinical practice. Secondary mutation in a kinase domain of the targeted kinase gene^30^ and reactivation of the targeted pathway based on an additional genomic alteration^31^ have been reported as mechanisms of acquired resistance to kinase inhibitors. To prevent and overcome this problem, we focused on both fused genes *FGFR3* and *TACC3* in *FGFR3-TACC3* fusion-positive cancer because the kinase fusion protein may have not only kinase action but also other actions based on the non-kinase fused gene. TACC3 inhibitor, KHS101, was originally identified a small molecule that selectively induces a neuronal differentiation phenotype. After screening for a specific target of KHS101, TACC3 was identified that had physically interacts with KHS101^20^. KHS101 has a potential to target coiled-coil domain of TACC3^32^, Therefore, we estimated that KHS101 has suppressed FGFR3-TACC3 fusion protein that retains coiled-coil domain of TACC3. Indeed, our results show that KHS101^20^ could suppress the FGFR3-TACC3 fusion protein and that dual inhibition of FGFR and TACC3 synergistically suppressed cell proliferation. However, when we assess the effect of TACC3 inhibitor (KHS101) to FGFR3-TACC3 fusion-transfected cancer cell lines, we need to pay attention to specificity of KHS101 for TACC3. Affinity-based purification methodology revealed physical interaction of KHS101 with TACC3^20^, although binding of KHS101 to other cellular proteins cannot be excluded.

In conclusion, our study suggested a novel fusion-specific treatment strategy against *FGFR3-TACC3* fusion-positive cervical cancer. Dual inhibition of FGFR and PI3K/AKT pathways may be a meaningful treatment strategy for other types of cancer that harbor both FGFR3-TACC3 fusion and genomic alteration of the PI3K/AKT pathway.

## Materials and methods

### TCGA data analysis

RNA sequencing data and clinical data from 306 TCGA uterine cervical cancer samples were downloaded from the Cancer Genome Hub and TCGA Data Portal, respectively. To identify high confidence fusion transcripts, we applied PRADA^8^ to the TCGA cervical cancer dataset. Fusion transcripts were extracted as previously reported^6^. For *FGFR3-TACC3* fusion-positive cervical cancer samples, mutation and copy number alteration data were also downloaded from the cBio portal^33^.

### Cell culture

Two human cervical epithelial cell lines (Ect1/E6E7 and End1/E6E7) and SiHa were purchased from the American Type Culture Collection (Rockville, MD, USA). HeLa, ME180, and Ca ski were purchased from the Japanese Collection of Research Bioresources Cell Bank (Osaka, Japan). Ect1/E6E7 and End1/E6E7 were cultured in keratinocyte-serum-free medium (Thermo Fisher Scientific, Waltham, MA USA) supplemented with 44.1 mg/l calcium chloride (final concentration 0.4 mM), 0.1 ng/ml human recombinant EGF, 0.05 mg/ml bovine pituitary extract, 50 IU/ml penicillin, and 50 μg/ml streptomycin. All cervical cancer cell lines were cultured in Dulbecco’s modified Eagle’s medium, supplemented with 10% fetal bovine serum, 50 IU/ml penicillin, and 50 μg/ml streptomycin. All cell lines were authenticated and tested for mycoplasma contamination before we used.

### Clinical samples

Frozen tumor tissues used in this study were obtained from 103 Japanese patients who were diagnosed with cervical cancer at the Niigata University, Tottori University, or Ryukyus University. Clinical characteristics of these patients are shown in Supplementary Table S6. The institutional review board at all sites approved this study (No. 682 and 837 in Niigata University, No. G158 in Tottori University, and No. 106 in University of the Ryukyus). All patients provided written informed consent for the collection of samples and subsequent analysis. All experiments were performed in accordance with approved guidelines and regulations.

### RT-PCR and Sanger sequencing

RT-PCR and Sanger sequencing were performed as previously reported^34^. In brief, total RNA was extracted from frozen samples using TRIzol (Invitrogen, Carlsbad, CA, USA) and from cells using the miRNeasy Mini kit (Qiagen, Tokyo, Japan). Total RNA (1 µg) was reverse-transcribed into cDNA using Prime Script II Reverse Transcriptase (Takara Bio, Shiga, Japan). cDNA (corresponding to 10 ng total RNA) was subjected to PCR amplification using KAPA Taq DNA polymerase (KAPA Biosystems, Woburn, MA, USA). The reactions were carried out in a thermal cycler under the following conditions: 40 cycles at 95°C for 30 s, at 60°C for 30 s, and at 72°C for 1 min, with a final extension at 72°C for 1 min.

PCR products were extracted and purified by NucleoSpin Gel and PCR Clean-up (Takara Bio), and were sequenced on an ABI 3130xl DNA Sequencer (Applied Biosystems, Foster City, CA, USA) using a BigDye Terminator kit (Applied Biosystems). The PCR primers used in this study are shown in Supplementary Table 7.

### Quantitative real-time RT-PCR

Quantitative real-time RT-PCR was performed with a Thermal Cycler Dice Real-Time System TP800 2.01C (Takara Bio). cDNA (corresponding to 10 ng of total RNA) was subjected to real-time PCR amplification analysis using SYBR Premix Ex Taq II (Takara Bio). The relative quantification method was used to measure the amounts of the respective genes normalized to *ACTB*. All primers used are shown in Supplementary Table 7.

### Cloning

cDNA of the full-length *FGFR3-TACC3* fusion transcripts (“Fusion 1” and “Fusion 2”), wild-type *FGFR3* isoform 3, and wild-type *TACC3* were isolated from fusion-positive tumor samples and amplified by RT-PCR using KOD or KOD-plus polymerase (Toyobo, Tokyo, Japan), then digested with *Eco*RI–*Not*I (Fusion 1, Fusion 2, and wild-type *FGFR3*) or *Xba*I–*Not*I (wild-type *TACC3*). All cloned sequences were verified by Sanger sequencing. PCR products were digested with restriction endonuclease and ligated into the pMRX-IRES-puro vector. The pMRX-IRES-puro vector was kindly provided by Dr. S. Yamaoka (Tokyo Medical and Dental University, Japan). Expression constructs were transfected into Plat-E cells using polyethylenimine. After 48 h from plasmid transfection, retrovirus was extracted from culture supernatants. Retroviral transfection was performed in the presence of 8 μg/ml polybrene for 48 h. Following that, SiHa, HeLa, ME180, and Ca Ski cells were selected by using 0.5 μg/ml puromycin, and Ect1/E6E7 and End1/E6E7 cells were selected by using 0.25 μg/ml puromycin for 1 week, respectively.

### Mutagenesis

The kinase activity-deficient mutants were constructed by replacing tyrosine with phenylalanine at codons K508M in the *FGFR3-TACC3* (“Fusion 1” and “Fusion 2”) using a Prime STAR site-directed mutagenesis kit (Takara Bio). Primers used in mutagenesis are shown in Supplementary Table 7.

### Immunoblot analysis

Cells were lysed in TNE buffer (10 mM Tris [pH 7.5], 150 mM NaCl, 1 mM ethylenediaminetetraacetic acid [EDTA]) supplemented with 1% NP-40, protease inhibitor cocktail, and phosphatase inhibitor. Samples were separated using a NuPAGE system (Thermo Fisher Scientific) on 4–12% Bis-Tris gels in MOPS-SDS buffer, and then transferred to a polyvinylidene difluoride membrane. Antibodies were purchased from the indicated suppliers: FGFR3 (sc-13121, Santa Cruz; dilution ratio 1:200), TACC3 (sc-22773, Santa Cruz; dilution ratio 1:200), phosphorylated ERK (#4370, Cell Signaling Technology; dilution ratio 1:1000), ERK (#4695, Cell Signaling Technology; dilution ratio 1:1000), phosphorylated-AKT (#4060, Cell Signaling Technology; dilution ratio 1:1000), AKT (#4691, Cell Signaling Technology; dilution ratio 1:1000), phosphorylated-STAT3 (#9145, Cell Signaling Technology; dilution ratio, 1:1000), STAT3 (#9139, Cell Signaling Technology; dilution ratio 1:1000), and actin (MAB1501R, Merck Millipore Headquarters; dilution ratio 1:1000). Blots were then incubated with either anti-mouse or anti-rabbit horseradish peroxidase-conjugated secondary antibodies (#115-035-166 and #111-035-144, Jackson Immuno Research; dilution ratio 1:10,000), and visualized by chemiluminescence. Images have been cropped for presentation. Full size images are presented in Supplementary Figures 8–14.

### Colony formation

Colony formation assay was performed using a CytoSelect 96-well cell transformation assay kit (Cell Biolabs, Inc., San Diego, CA, USA). In brief, cells were plated in soft agar in a 96-well plate at 1000 cells/well (SiHa, ME180, HeLa and Ca Ski) at 5000 cells/well (Ect1/E6E7 and End1/E6E7), and cultured for 7–10 days (SiHa, HeLa, ME180, and Ca Ski) and 14 days (Ect1/E6E7 and End1/E6E7). The transformation was determined according to the manufacturer’s protocol. Data represent the mean ± the standard deviation (S.D.) of three independent assays.

### Xenograft establishment

Cells (*n* = 2 × 10^6^) were dissociated into single cells with trypsin/EDTA (Gibco, Thermo Fisher Scientific), suspended in 100 μl culture medium containing 50% Matrigel (BD Biosciences), and used for subcutaneous injection into the flanks of NOG mice (NOD/Shi-scid, IL-2Rγ^null^; 6-week-old females; Central Institute for Experimental Animals, Kawasaki, Japan) with a 27-gauge needle. Mice were monitored every 3–4 days until 3 or 4 weeks in SiHa and ME180 cells and 105 days in Ect1/E6E7 cells (*FGFR3-TACC3* fusion or control vector-transfected cells). No mice randomization was performed and no blinding procedures were performed. All animal experiments and protocols were approved by the Animal Care and Use Committees of Niigata University and performed in accordance with institutional policies. For xenograft establishment, 6 mice for SiHa and ME180 cells and 10 mice for Ect1/E6E7 cells were used. After sacrificing the mice, tumor volume and weight were determined.

### Histological analysis

Xenograft tumors were fixed in neutral formalin, embedded in paraffin, and stained with hematoxylin and eosin. All histological specimens were reviewed by a gynecology pathologist (T.M.). In addition, immunohistochemical staining for the FGFR3 protein was performed, as previously reported^35^. Briefly, after deparaffinization, antigen retrieval was carried out with Target Retrieval Solution (10 mM citrate buffer, pH 6.0; Dako) in a microwave for 30 min at 96°C. Subsequently, the sections were incubated overnight with primary antibody (sc-13121, Santa Cruz; dilution ratio 1:50) at 4°C and biotinylated anti-mouse secondary antibodies (Vector Laboratories, Burlingame, CA, USA) were added, followed by incubation with ABC reagent (Dako) and 3,3′-diaminobenzidine (Sigma, St. Louis, MO, USA). Slides were counterstained with hematoxylin.

### RNA sequencing

We performed RNA sequencing for Ect1/E6E7, SiHa, and ME180 cell lines in which *FGFR3-TACC3* fusion, *FGFR3* kinase-dead fusion, and the control vector were transfected, respectively. Cell culture and RNA extraction were independently performed three times. The quantity and quality of the extracted RNA were evaluated with RNA 6000 Nano Assay Kit on the Agilent 2100 Bioanalyzer (Agilent Technologies, Santa Clara, CA, USA). We used samples for which the RNA integrity number was greater than or equal to 8.0. One microgram of the extracted total RNA was used for the library preparation, which was conducted by using a TruSeq Stranded mRNA Library Prep Kit (Illumina, San Diego, CA, USA) according to the manufacturer’s protocol. The modal size of the library was about 300 bp. The adapter-ligated cDNA was amplified with 12 cycles of PCR. The samples were sequenced on the Illumina HiSeq 2500 platform with the 2 × 100-bp paired-end read module.

We applied PRADA analysis to data from the above RNA sequencing experiments and obtained a list of fusion transcripts and gene expression data for each sample.

### Gene set enrichment analysis

We used gene expression data to perform GSEA between the *FGFR3-TACC3* fusion-transfected group and the control group or between the *FGFR3-TACC3* fusion kinase-dead transfected group and the control group. Biocarta pathway gene sets were downloaded from the Molecular Signatures Database (http://software.broadinstitute.org/gsea/msigdb). We used a normalized enrichment score and *q*-value to examine overrepresented pathways and extracted pathways for which the −log_2_ (*q*-value) was greater than 2.0.

### Inhibitor experiments

BGJ398 (Selleck Chemicals, Houston, TX, USA), MK2206 (Selleck Chemicals), trametinib (Selleck Chemicals), and KHS101 (SIGMA-Aldrich, St. Louis, MO, USA) were dissolved in dimethyl sulfoxide. Each cell line was seeded in six-well plates at 1.5 × 10^5^ cells/well and was exposed to inhibitors for 24 h. Inhibitor-treated cell lines were lysed and used for immunoblot assays.

For the cell proliferation assay, each cell was seeded in 96-well plates at 1000 cells/well and exposed to inhibitors for 24 h. Cell proliferation was measured after 72 h for each inhibitor using the CellTiter Glo assay (Promega, Mannheim, Germany) according to the manufacturer’s protocols. The data represent the mean ± S.D. of four independent assays. One and two asterisks denote significant *p*-values, at 5% and 1%, respectively.

### Enzyme-linked immunosorbent assay

SiHa and ME180 cells (2 × 10^5^ cells/well) were seeded in 1 ml of Dulbecco’s modified Eagle’s medium, supplemented with 10% fetal bovine serum, 50 IU/ml penicillin, and 50 μg/ml streptomycin in six-well plates and cultured for 24 h. The secretion of IL-8 by the supernatant of SiHa and ME180 cells was detected by Human CXCL8/IL-8 Quantikine ELISA Kit (R&D Systems, Minneapolis, MN, USA), according to the manufacturer’s instructions. For the inhibitor assays, 1 × 10^5^ cells/well were seeded in 1 ml of the same medium and exposed to inhibitors for 48 h.

### Whole-exome sequencing and analysis

Extracted DNA was quantified with a Qubit dsDNA HS Assay Kit (Thermo Fisher Scientific). Two hundred nanograms of DNA were sheared on a Covaris S2 (Covaris, Woburn, MA, USA) into fragments with a modal length of 350–500 bp. Sequencing libraries with different indexed adapters were constructed with SureSelect XT Reagent Kits (Agilent). Target enrichment was conducted with SureSelect Human All Exon V5+lncRNA Kit (Agilent) according to the manufacturer’s protocol. The libraries were sequenced on an Illumina HiSeq 2500 platform in rapid run mode with a 2 × 100-bp paired-end module (Illumina).

As a quality control step, the Illumina adapter and low-quality sequences were trimmed using Trimmomatic, version 0.32^36^. When DNA inserts were shorter than the read length, the non-adapter portion of the forward and reverse sequences became reverse complements, i.e., the reverse read contained the same sequence information as the forward read. In such cases, the forward read was retained to avoid doubling the count bases from overlapping alignments of paired-end reads. The paired-end and single-end read datasets were separately aligned to a human reference genome (hg19) by BWA, version 0.7.12^37^, and merged for subsequent analyses by SAMtools, version 0.1.19^38^. The aligned reads were processed for removal of PCR duplicates by Picard Tools, version 1.111 (broadinstitute.github.io/picard). Local realignment and base quality recalibration were implemented by GATK, version 3.2.2^39,40^. Averages of depth and coverages over target regions captured by SureSelect Human All Exon V5+lncRNA Kit were calculated by DepthOfCoverage and CallableLoci tools in GATK, respectively^39,40^.

### Detection of putative somatic mutations

We used an analytical pipeline in which putative somatic single-nucleotide variants (SNVs) and short insertions and deletions (indels) were called based solely on whole-exome sequencing data from tumor samples without matched normal samples. SNVs and indels were determined based on whole-exome sequencing data from tumor-derived DNA by using HaplotypeCaller and VariantRecalibraor of GATK^39,40^. Functional annotation of the identified variants was implemented by ANNOVAR^41^. We hypothesized that somatic mutations were not merely identified as germline genetic variations in the general population. We defined putative somatic SNVs and indels as variants for which the allele frequencies were less than 0.1% in all populations, based on publicly available databases provided by the following whole-genome and -exome sequencing projects: the 1000 Genomes Project, the Exome Aggregation Consortium, and the Human Genetic Variation Database^42–44^. Prevalence of putative somatic mutations from previous genome-wide screenings in various cancer types was retrieved from COSMIC, release v79^45^.

### Detection of putative somatic copy number alterations

We sought putative somatic copy number alterations by using Control-FREEC software^46^. The read counts per region covered by consecutive capture probes were normalized by GC content. We excluded the regions with low mappability scores calculated for the read length of 100-bp, allowing up to two mismatches. Since matched normal DNA samples were not available in this study, the read counts of each tumor sample were compared with “reference read counts” obtained by pooling blood-derived sequencing data generated by the same exome sequencing platform from 21 women without any history of gynecological cancers.

### Statistical analysis

Data were expressed as the mean ± S.D. The unpaired *t*-test or Fisher’s exact test were used to evaluate the significance between data groups. *p*-Values less than 0.05 indicated statistical significance.

## Electronic supplementary material

Supplementary Information
