# Supplementary material for: Novel therapeutic strategy for cervical cancer harboring FGFR3-TACC3 fusions
Source: Oncogenesis. 2018 Jan 23;7(1):4. doi: 10.1038/s41389-017-0018-2 (PMC5833787; doi:10.1038/s41389-017-0018-2)
Supplement: Supplementary file 1 — Supplementary Information [file 41389_2017_18_MOESM1_ESM.pdf]

Supplementary Information

Novel therapeutic strategy for cervical cancer harboring *FGFR3-TACC3* fusions

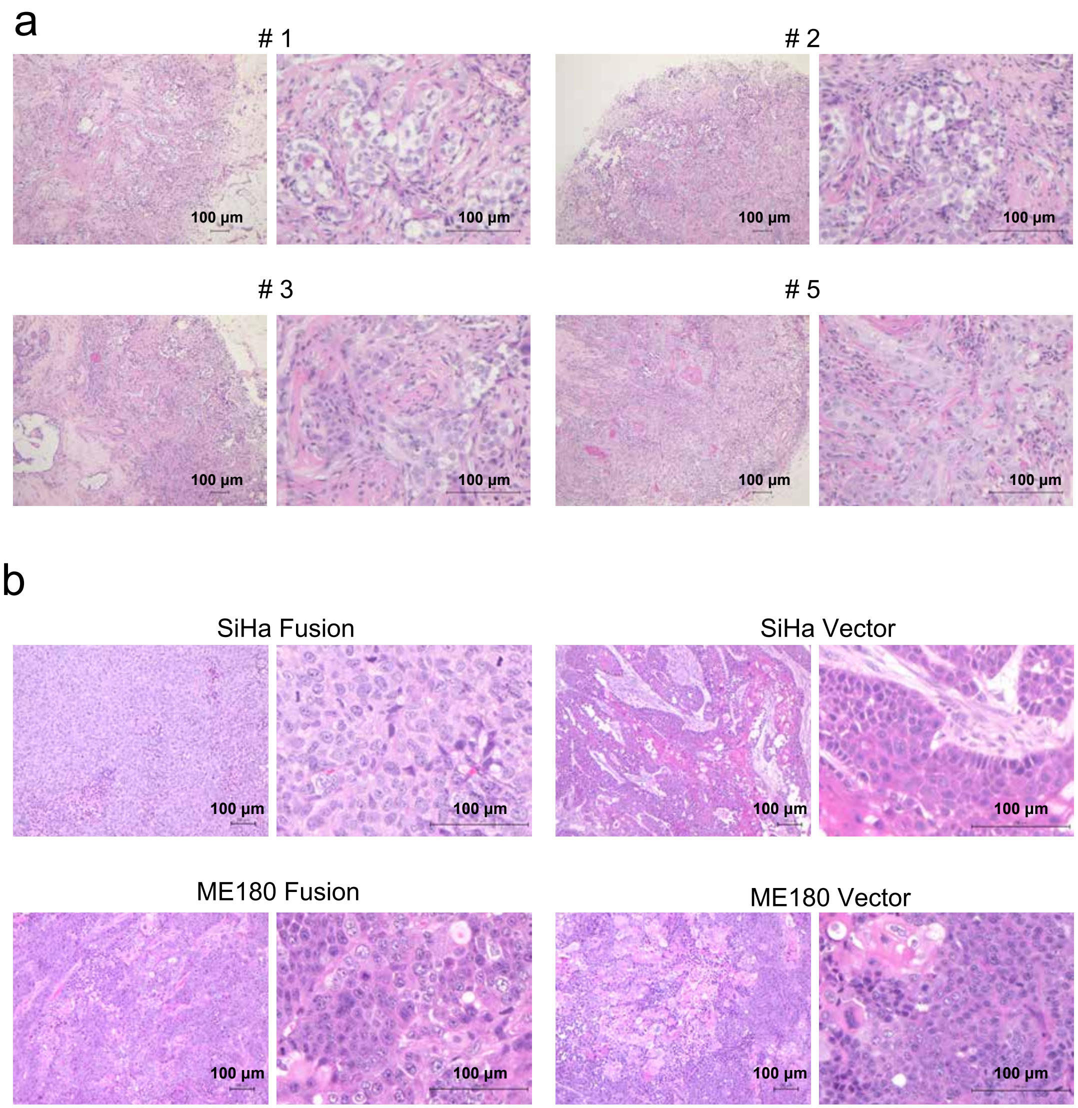
a
1
2
3
5
100 μm
b
SiHa Fusion
SiHa Vector
ME180 Fusion
ME180 Vector

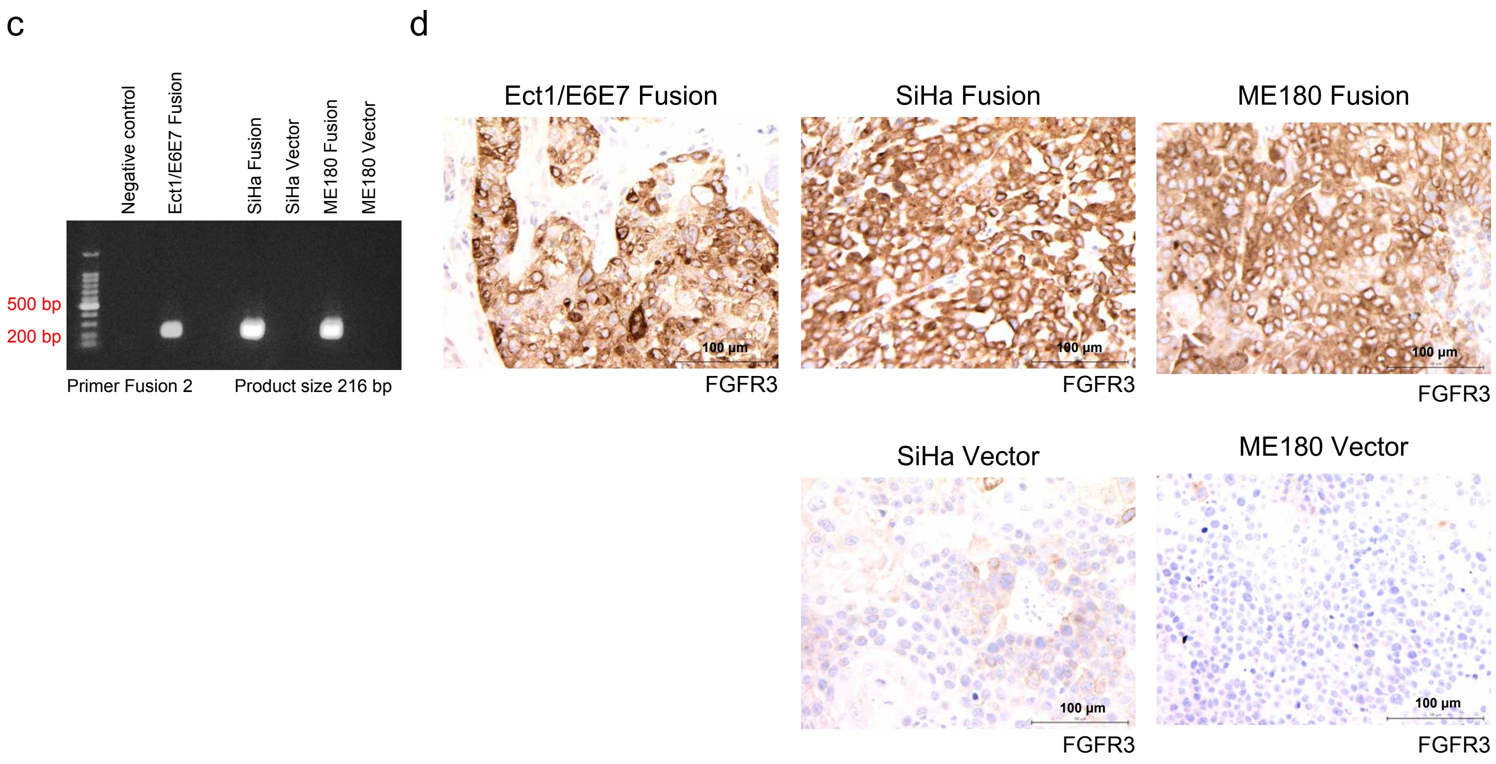

Supplementary Figure 1.
Histological findings of xenograft tumors derived from *FGFR3-TACC3* fusion-transfected Ect1/E6E7, SiHa and ME180. **(a)** Representative images of H&E stained xenograft tumors derived from *FGFR3-TACC3* fusion-transfected Ect1/E6E7 (100X and 400X magnification). All tumors were histologically diagnosed as squamous cell carcinoma. **(b)** Representative images of H&E stained xenograft tumors derived from *FGFR3-TACC3* fusion-transfected and control vector-transfected SiHa and ME180 cell lines (100X and 400X magnification). Tumors derived from *FGFR3-TACC3* fusion-transfected SiHa or ME180 cells demonstrated obvious reduction of keratinizing area compared to the controls. **(c)** Confirmation of the *FGFR3-TACC3* fusion transcript in xenograft tumors based on RT-PCR. **(d)** Immunohistochemical staining for xenograft tumors showed FGFR3 overexpression in xenograft tumors derived from *FGFR3-TACC3* fusion-transfected Ect1/E6E7, SiHa, and ME180 cell lines (200X magnification; scale bar, 100 μm).

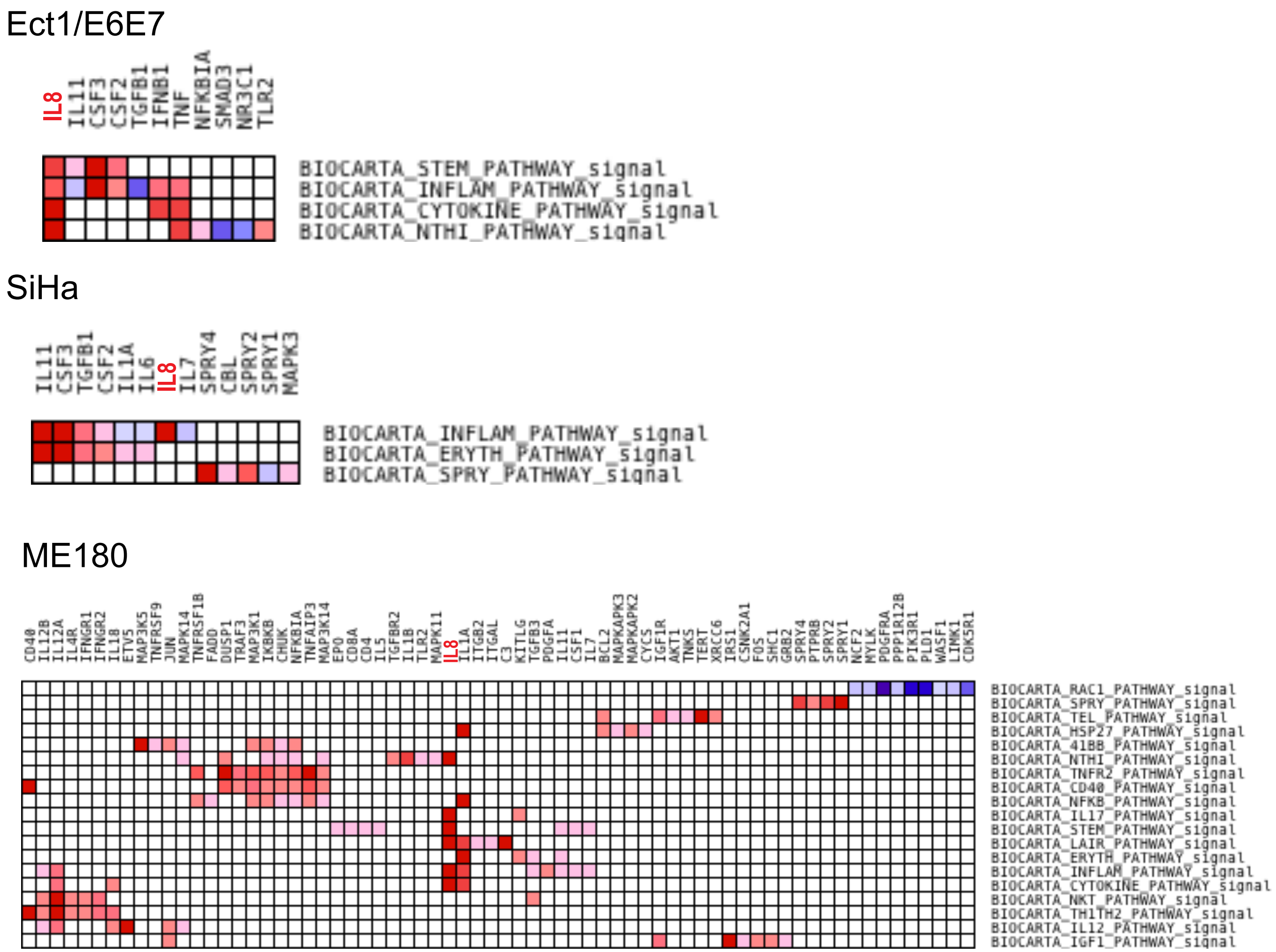

Supplementary Figure 2.
Leading edge analysis of significant GSEA gene sets in *FGFR3-TACC3* fusion-transfected cell lines (Ect1/E6E7, SiHa and ME180).
Leading edge analysis demonstrated that overexpression of *IL-8* was common among *FGFR3-TACC3* fusion-transfected Ect1/E6E7, SiHa, and ME180 cell lines.

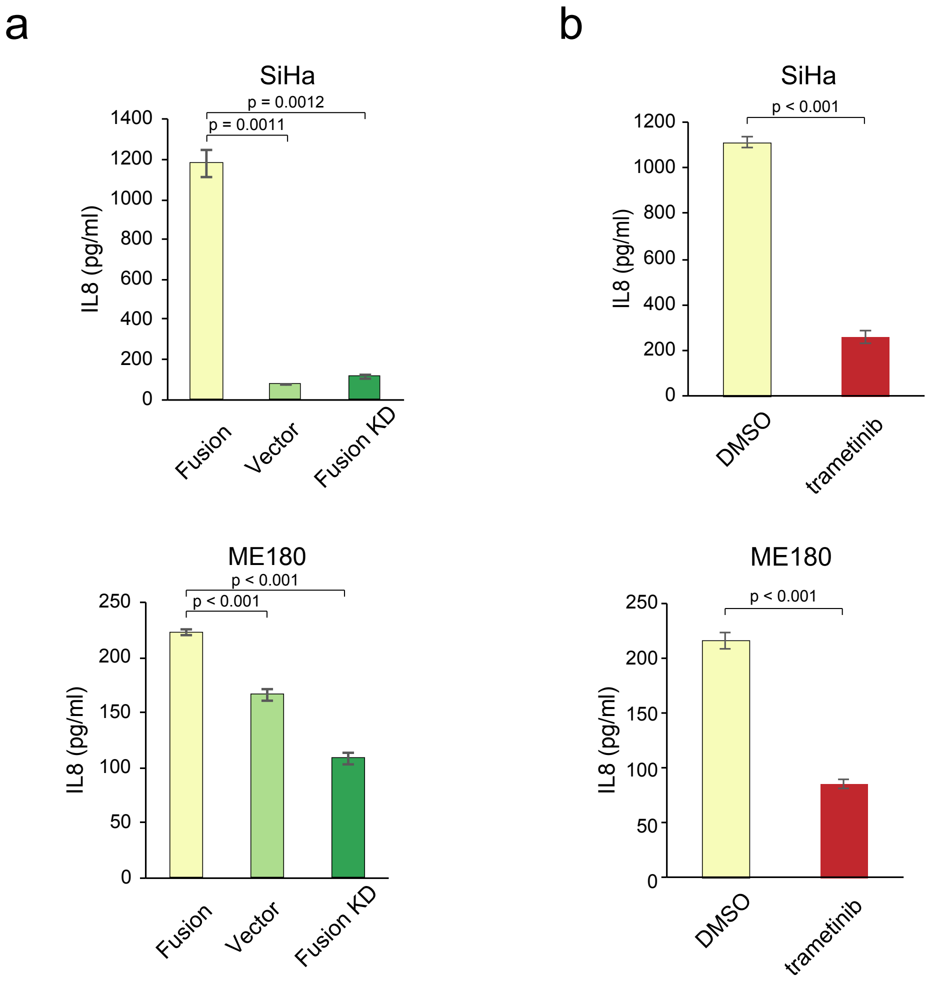

Supplementary Figure 3.
Expression of the *FGFR3-TACC3* fusion gene induces IL-8 secretion via MAPK pathway activation.
(a) Secretion of IL-8 was assessed by ELISA assay 24 hours after cell seeding.
(b) Secretion of IL-8 was assessed after treatment with trametinib (0.1 μg/ml) for 48 hours (** denotes $p < 0.01$, unpaired t-test)

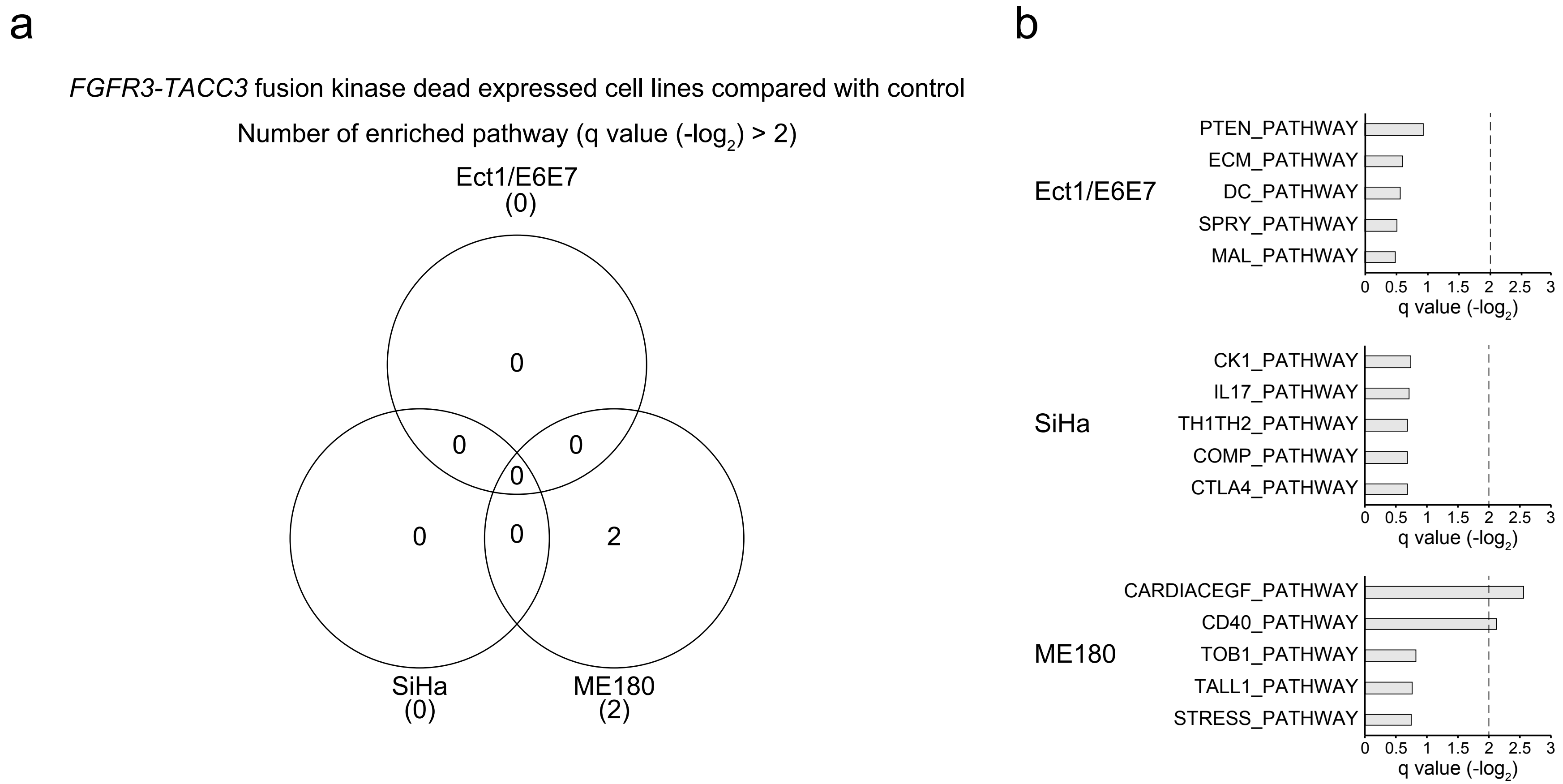

Supplementary Figure 4.
No common overrepresented pathway was present among the *FGFR3-TACC3* KD fusion group.
(a) Venn diagram shows no common overrepresented pathway among *FGFR3-TACC3* kinase dead domain (KD) fusion-transfected Ect1/E6E7, SiHa, and ME180 cell lines.
(b) Five top-ranked overrepresented pathways based on GSEA between the *FGFR3-TACC3* KD fusion group and control group.

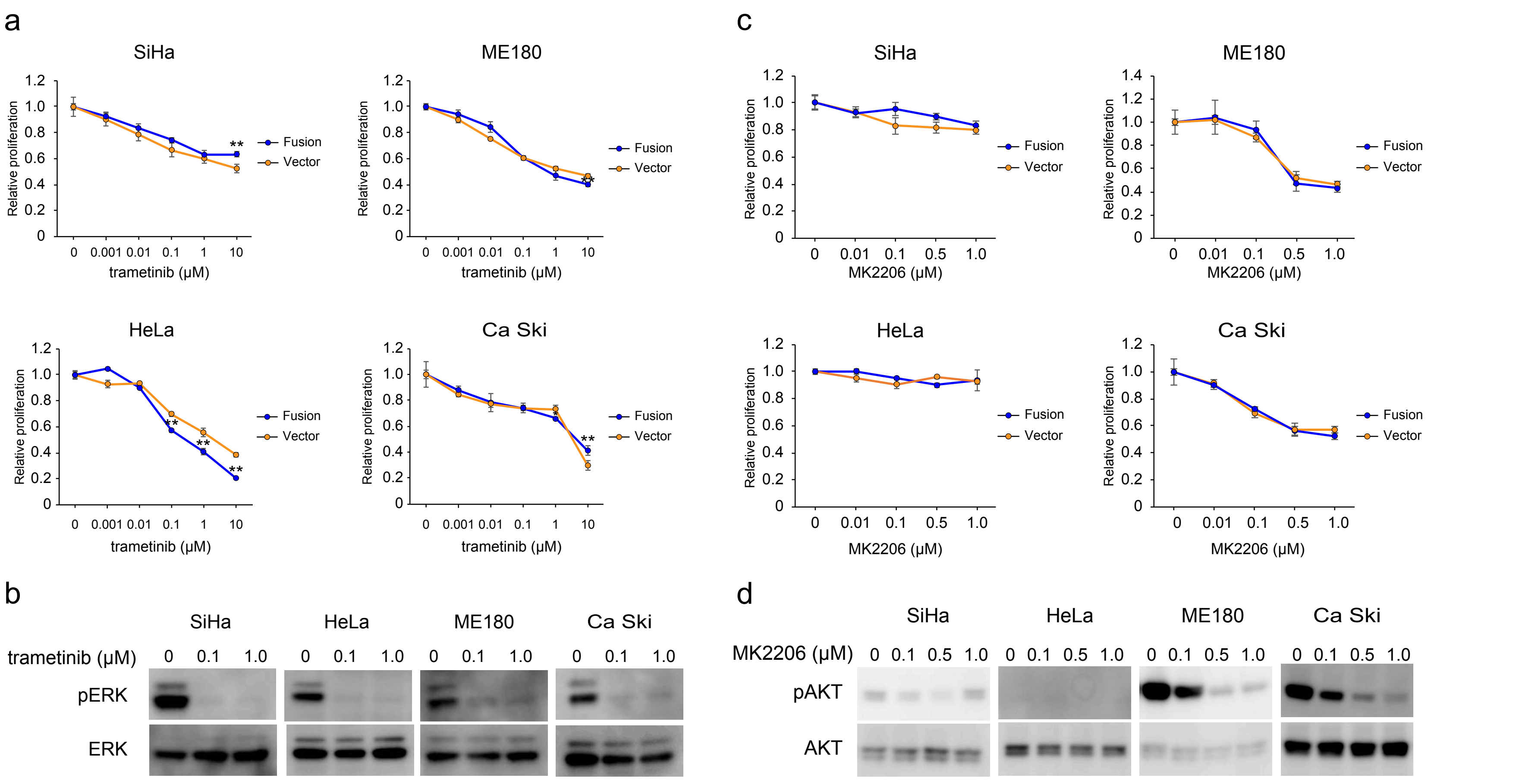

Supplementary Figure 5.

Effectiveness of a MEK inhibitor or AKT inhibitor in *FGFR3-TACC3* fusion-transfected cell lines.

(a, c) Cell proliferation was assessed by the Cell Titer Glo Assay after 72 hours of treatment with a MEK inhibitor (trametinib) or AKT inhibitor (MK2206). Data represent the average and standard deviation of quadruplicate experiments. Relative proliferation at each concentration was tested by unpaired t-test (** denotes $p < 0.01$). **(b, d)** Western blot analysis of *FGFR3-TACC3* fusion-transfected cell lines (SiHa, HeLa, ME180, and Ca Ski) after 24 hours of treatment with a MEK inhibitor (trametinib) or AKT inhibitor (MK2206).

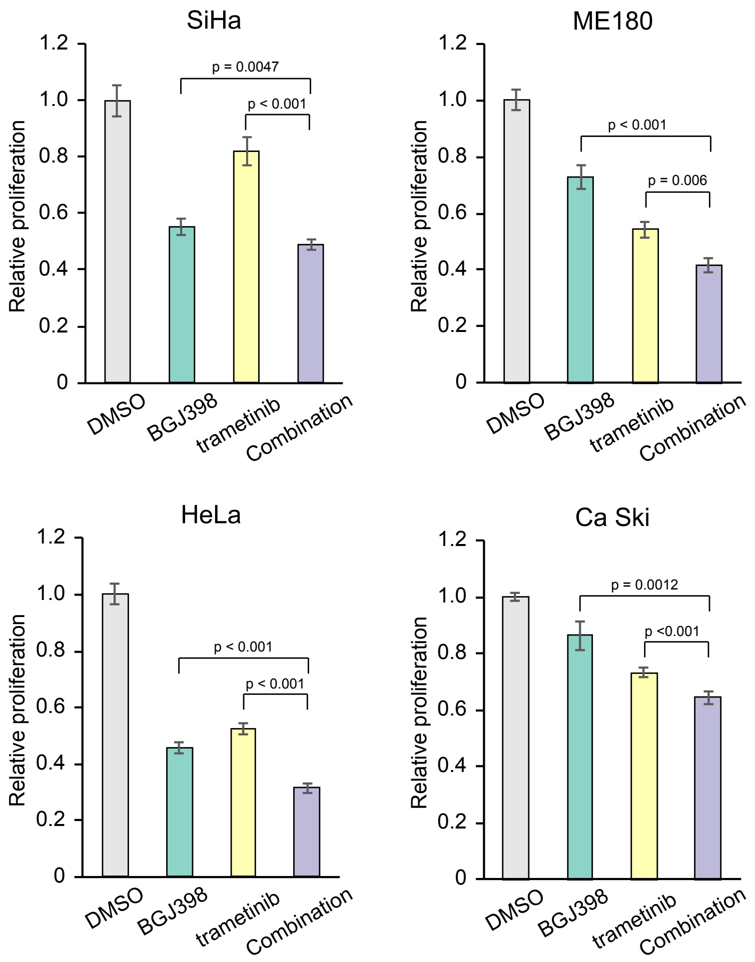

Supplementary Figure 6.
Suppression of cell proliferation of *FGFR3-TACC3* fusion-transfected cell lines after treatment with FGFR and/or MEK inhibitors.
Cell proliferation was assessed by the Cell Titer Glo Assay after 72 hours of treatment with BGJ398 (0.1µg/ml), trametinib (0.1µg/ml), or the combination of BGJ398 and trametinib. Bar graph represents the average ± standard deviation of quadruplicate experiments.

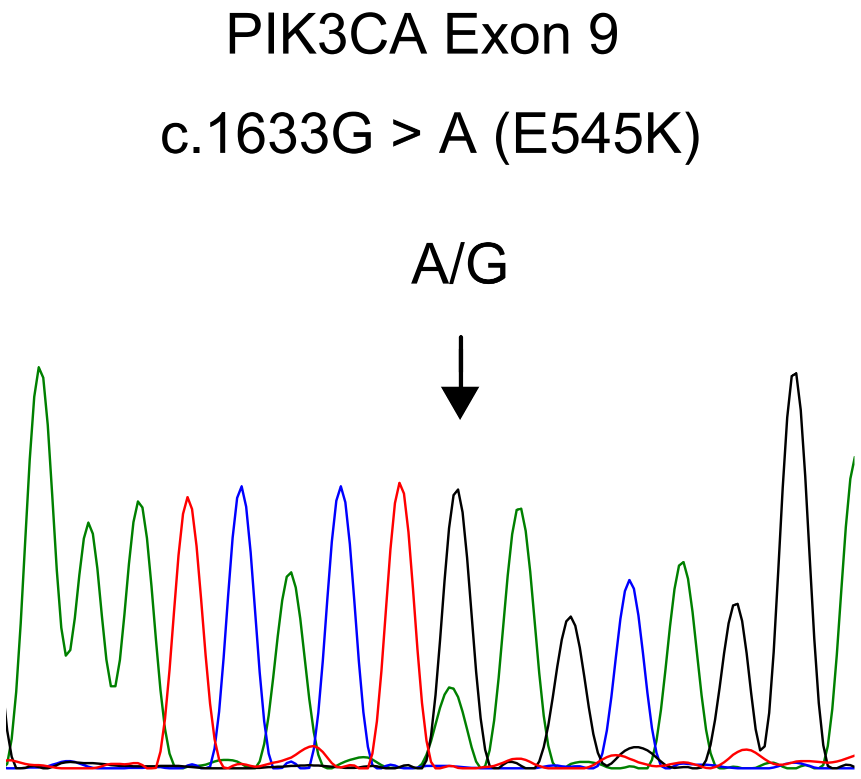

Supplementary Figure 7.
Validation of the *PIK3CA* activation mutation in the “Fusion 1”-positive cervical cancer sample.
Electropherogram from Sanger sequencing of *PIK3CA*. Arrow shows the two overlapping peaks of the mutated site.

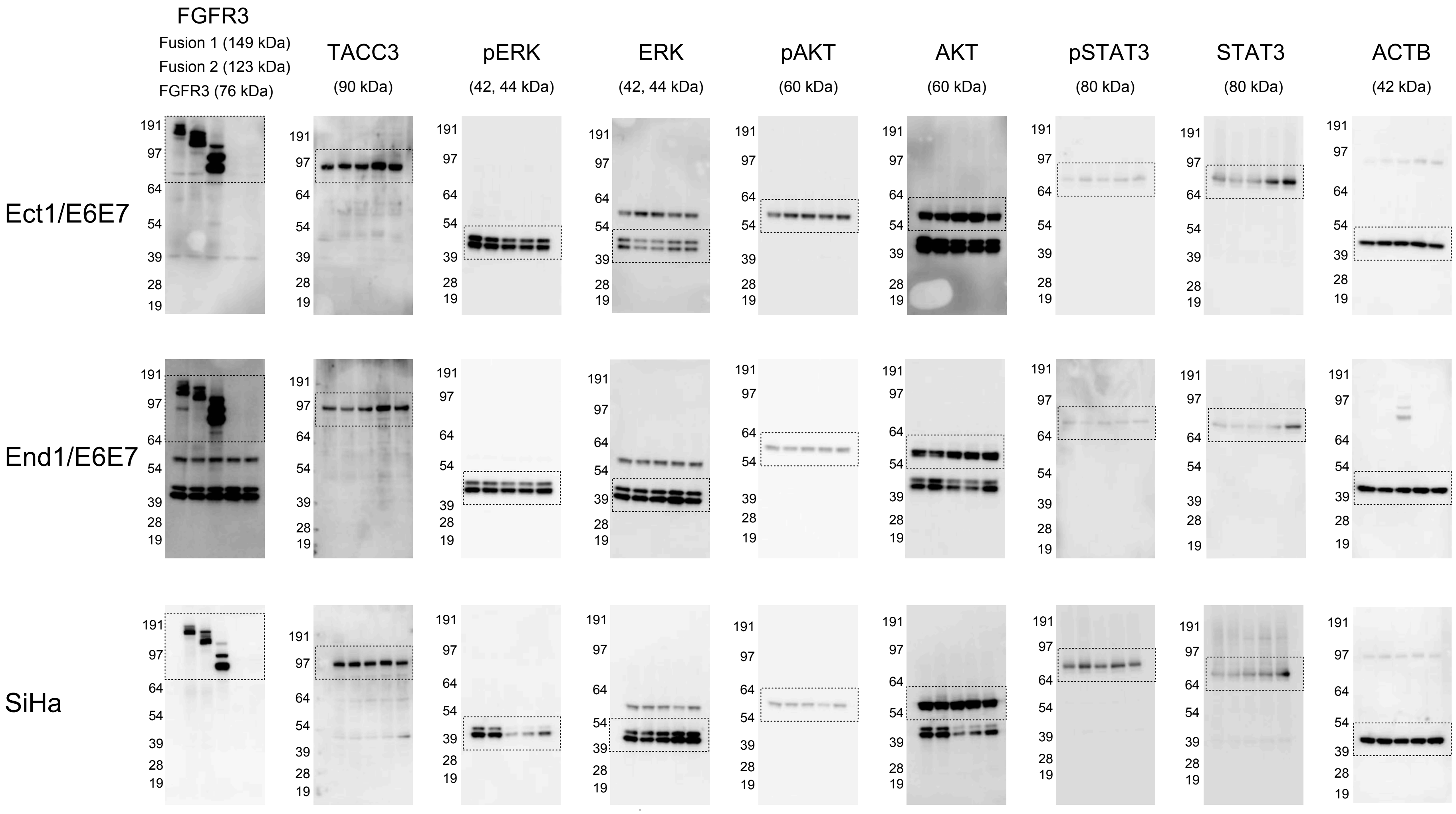

Supplementary Figure 8.
Full Western blot images used in Figure 2b and 2e

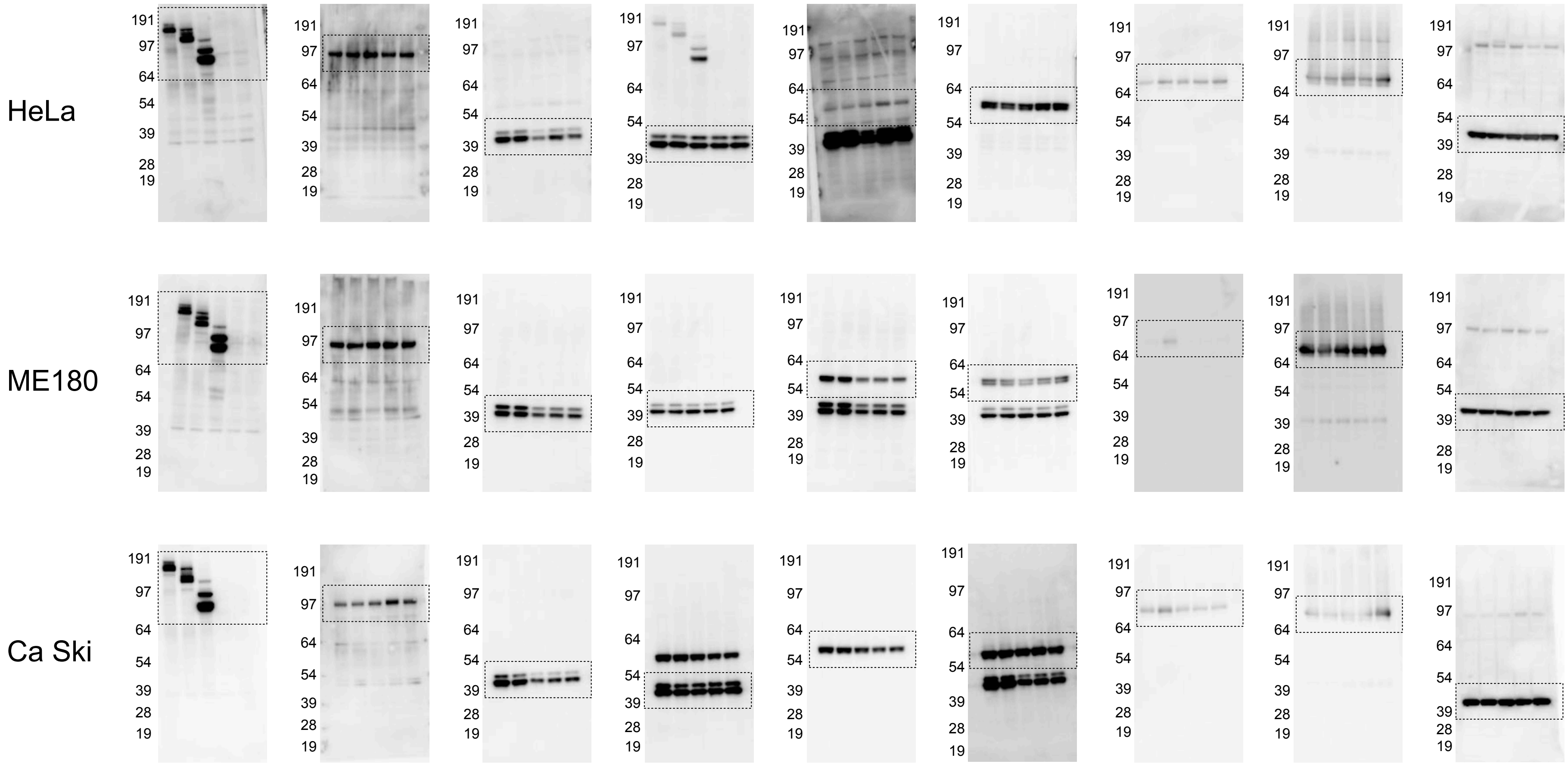

Supplementary Figure 8. (continued)

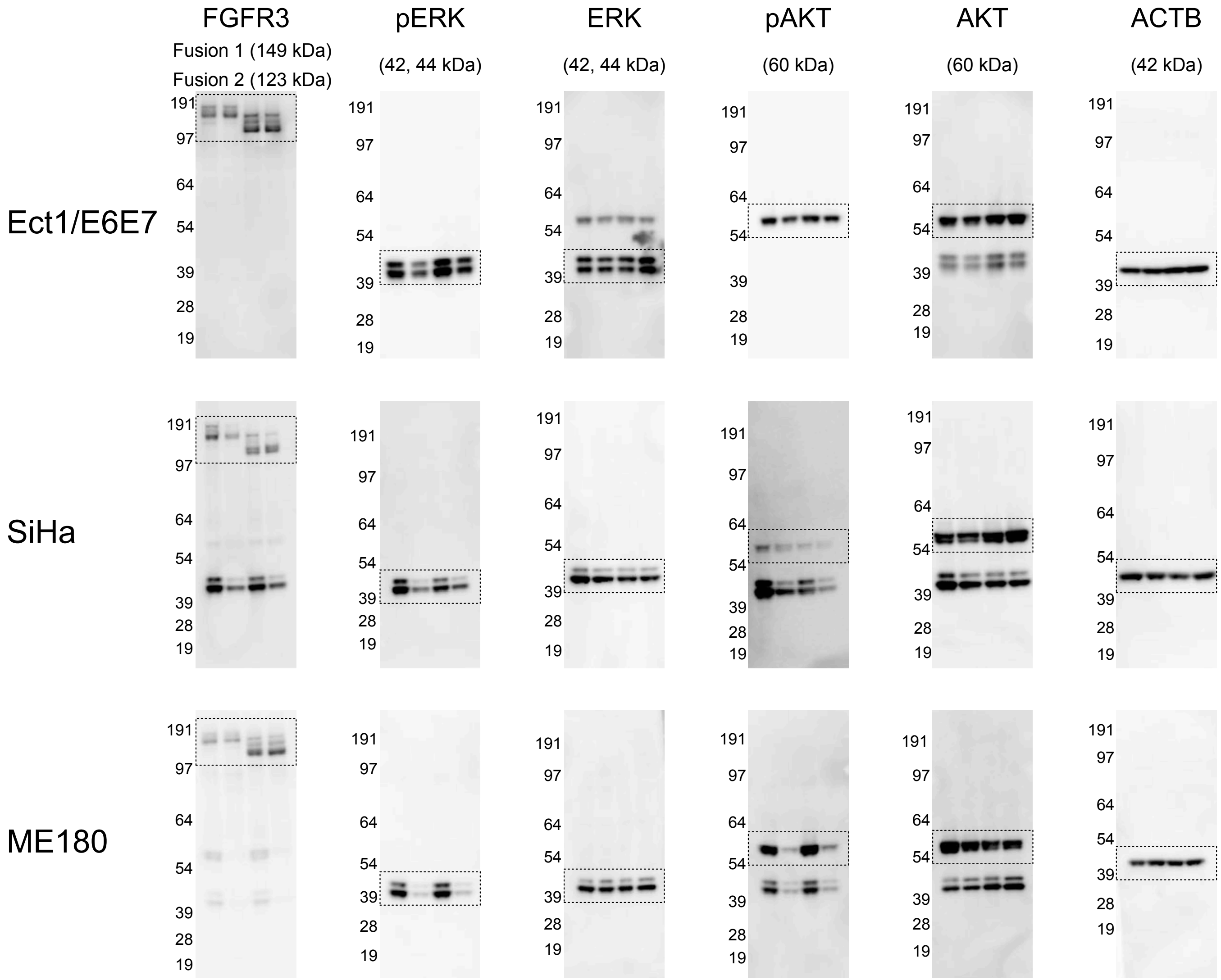

Supplementary Figure 9.
Full Western blot images used in Figure 3a.

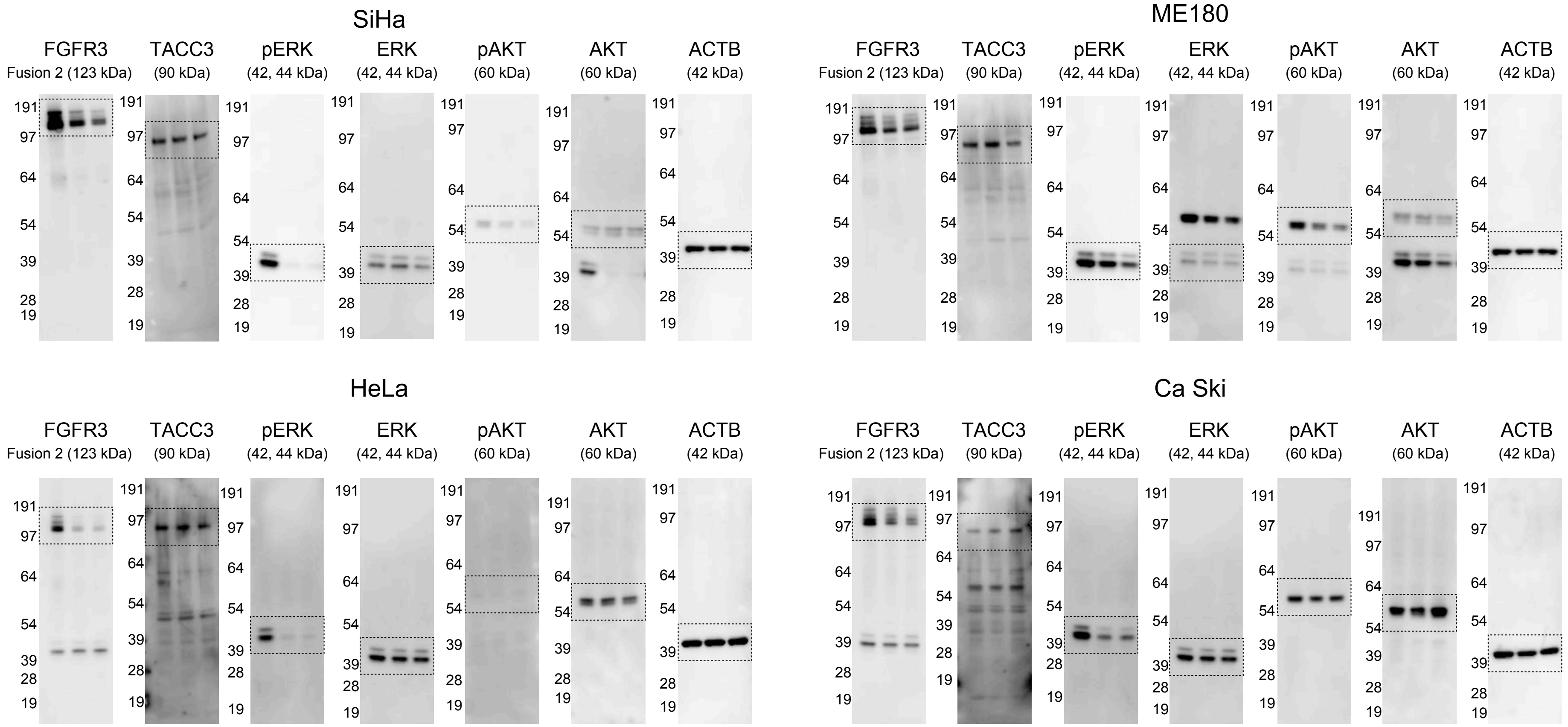

Supplementary Figure 10.
Full Western blot images used in Figure 4c

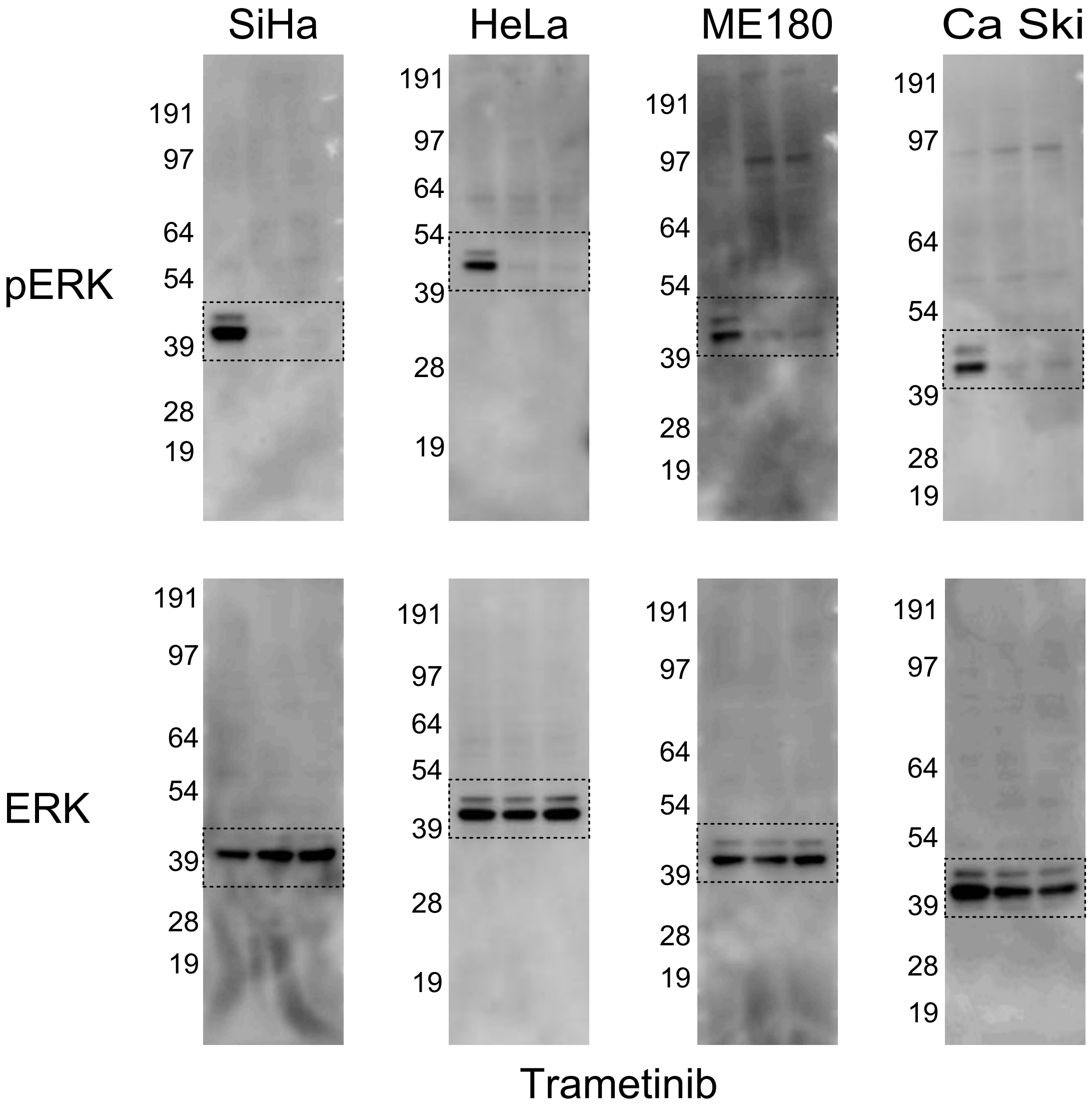

Supplementary Figure 11.
Full Western blot images used in Supplementary Figure 5b

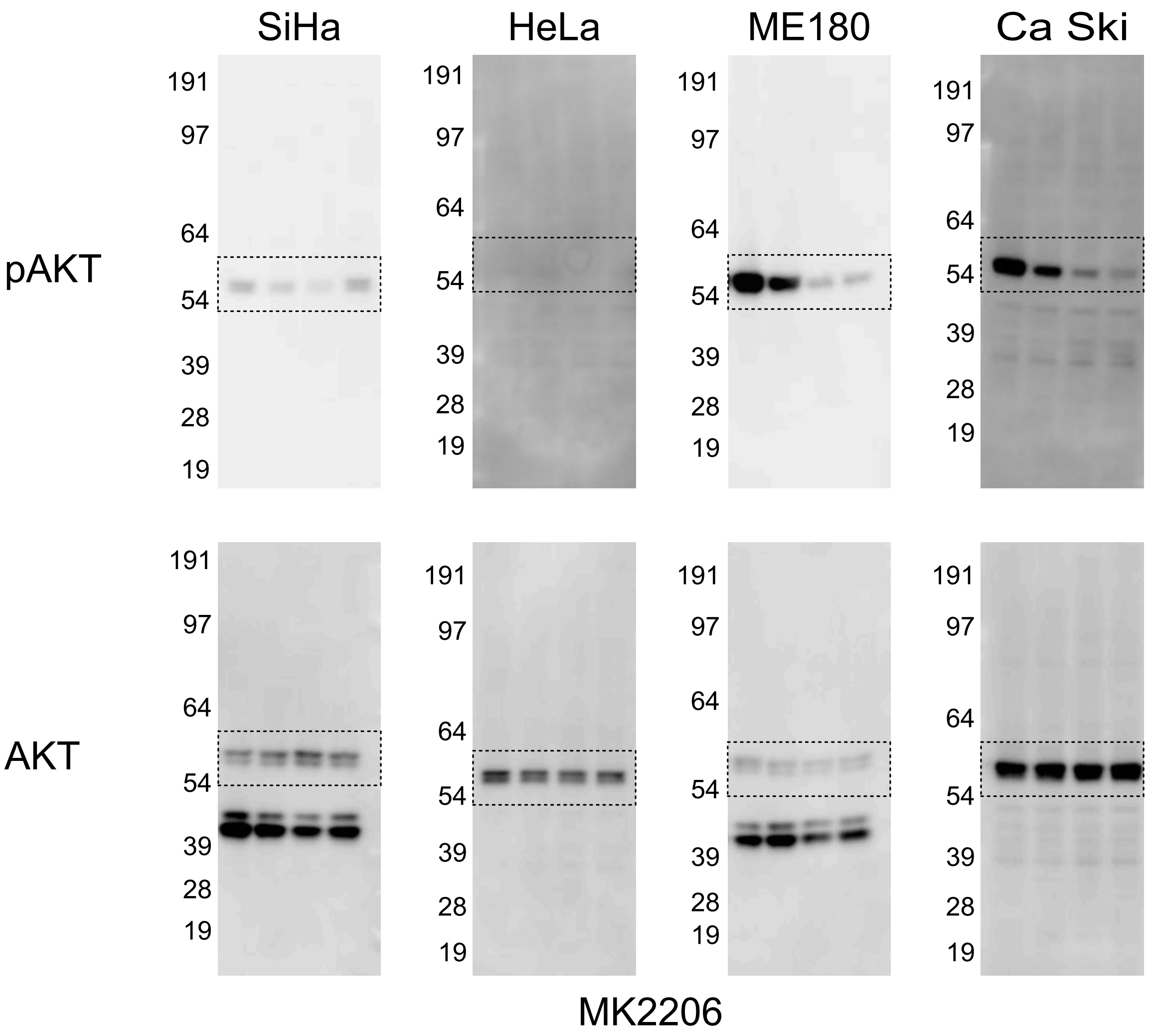

Supplementary Figure 12.
Full Western blot images used in Supplementary Figure 5d

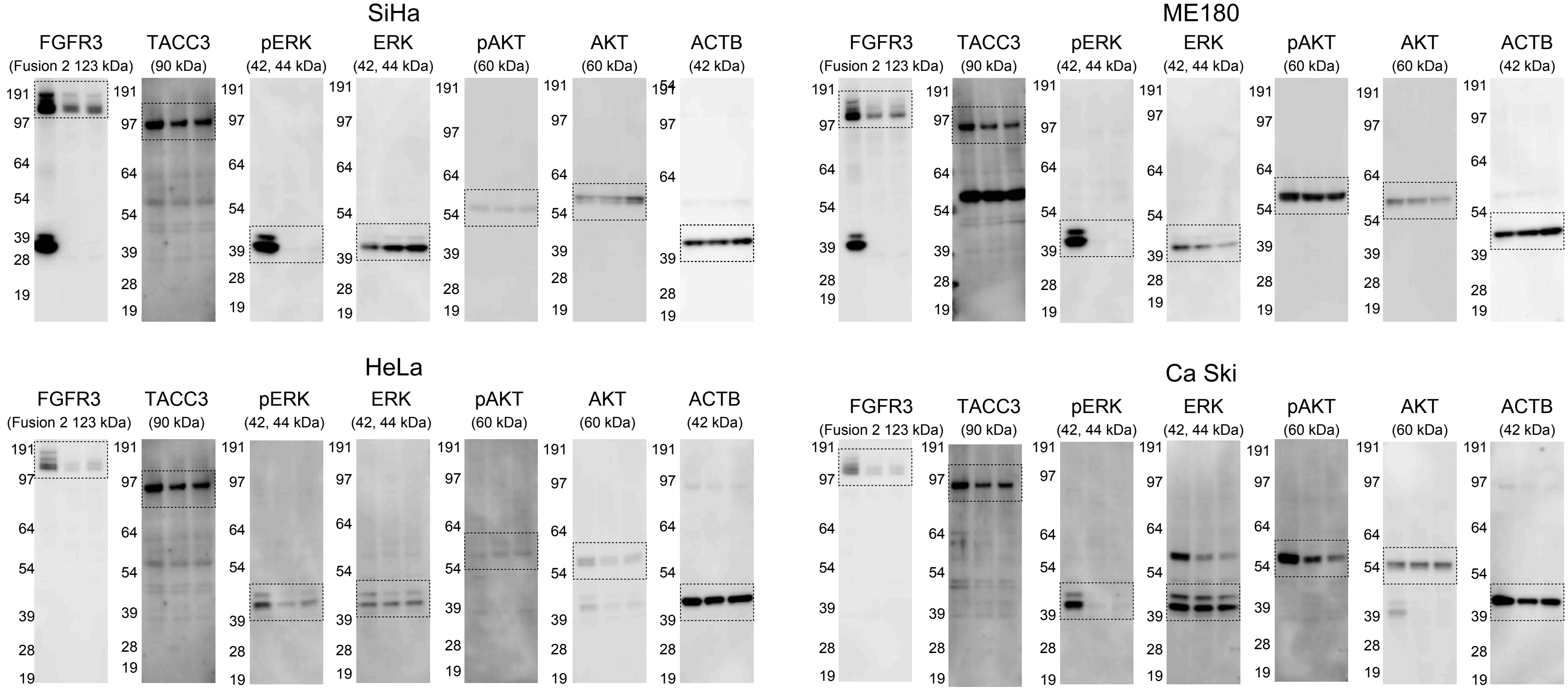

Supplementary Figure 13.
Full Western blot images used in Figure 6a

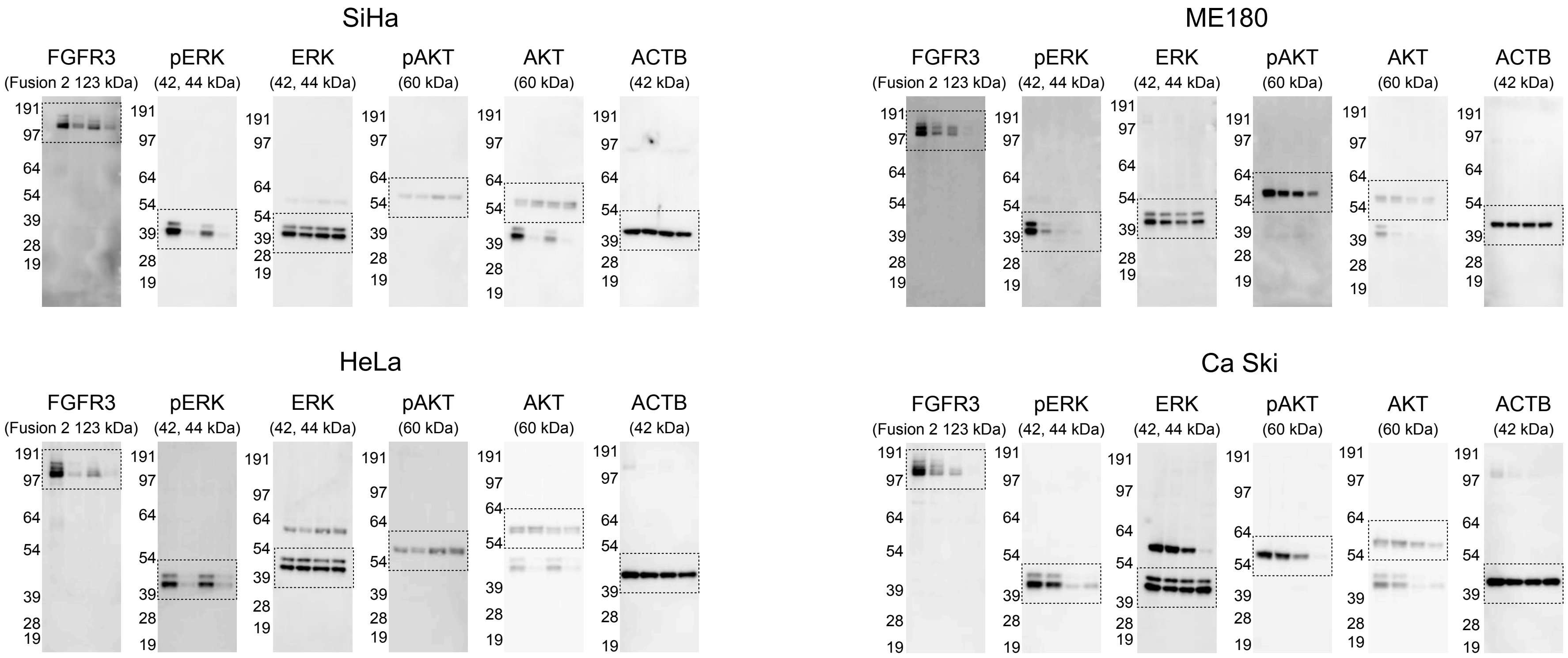

Supplementary Figure 14.
Full Western blot images used in Figure 6c

	Squamous cell carcinoma	Adenocarcinoma	p -value
Lung Cancer	1.0 % (5/502)	0 % (0/541)	0.026
Cervical Cancer	1.6 % (4/253)	0 % (0/47)	0.60

Supplementary Table 1.
Different frequency of *FGFR3-TACC3* fusion events between squamous cell carcinoma and adenocarcinoma in the TCGA lung and cervical cancer datasets

	SiHa	HeLa	ME180	Ca Ski	Role in cancer
PIK3CA	wild	wild	p.E545K	p.E545K	Oncogene
INSM2	wild	wild	p.P1855fs*21	p.Q4079*	NA
KMT2D	wild	wild	p.P1585fs*21	p.Q3809*	NA
MLL2	wild	wild	p.T56N	p.A304E	NA
RYR1	wild	wild	p.R777H	p.E813G	NA
WDR27	wild	wild	p.T1155I	p.V895L	NA
BAZ2A	p.C1073R	p.K714N	wild	wild	NA
CRB1	p.L1316S	p.P876L	wild	wild	NA
TRAPPC10	p.F223L	p.H1171R	wild	wild	NA
VPS13C	p.H131R	p.D1639H	wild	wild	NA

NA ; Not Applicable

Supplementary Table 2.
Genetic background of four cervical cancer cell lines based on the COSMIC database

SamleID	Gene A	Gene B	Discordant read pairs	Junction spanning reads	Junction
SiHa fusion 2_1	*FGFR3*	*TACC3*	324	866	FGFR3:4:1806661_TACC3:4:1732899
SiHa fusion 2_2	*FGFR3*	*TACC3*	349	875	FGFR3:4:1806661_TACC3:4:1732899
SiHa fusion 2_3	*FGFR3*	*TACC3*	280	776	FGFR3:4:1806661_TACC3:4:1732899
SiHa fusion 2KD_1	*FGFR3*	*TACC3*	215	464	FGFR3:4:1806661_TACC3:4:1732899
SiHa fusion 2KD_2	*FGFR3*	*TACC3*	197	449	FGFR3:4:1806661_TACC3:4:1732899
SiHa fusion 2KD_3	*FGFR3*	*TACC3*	151	300	FGFR3:4:1806661_TACC3:4:1732899
ME180 fusion 2_1	*FGFR3*	*TACC3*	183	342	FGFR3:4:1806661_TACC3:4:1732899
ME180 fusion 2_2	*FGFR3*	*TACC3*	162	276	FGFR3:4:1806661_TACC3:4:1732899
ME180 fusion 2_3	*FGFR3*	*TACC3*	246	550	FGFR3:4:1806661_TACC3:4:1732899
ME180 fusion 2KD_1	*FGFR3*	*TACC3*	135	441	FGFR3:4:1806661_TACC3:4:1732899
ME180 fusion 2KD_2	*FGFR3*	*TACC3*	158	452	FGFR3:4:1806661_TACC3:4:1732899
ME180 fusion 2KD_3	*FGFR3*	*TACC3*	176	462	FGFR3:4:1806661_TACC3:4:1732899
Ect1/E6E7 fusion 2_1	*FGFR3*	*TACC3*	227	800	FGFR3:4:1806661_TACC3:4:1732899
Ect1/E6E7 fusion 2_2	*FGFR3*	*TACC3*	242	721	FGFR3:4:1806661_TACC3:4:1732899
Ect1/E6E7 fusion 2_3	*FGFR3*	*TACC3*	187	519	FGFR3:4:1806661_TACC3:4:1732899
Ect1/E6E7 fusion 2KD_1	*FGFR3*	*TACC3*	119	398	FGFR3:4:1806661_TACC3:4:1732899
Ect1/E6E7 fusion 2KD_2	*FGFR3*	*TACC3*	132	388	FGFR3:4:1806661_TACC3:4:1732899
Ect1/E6E7 fusion 2KD_3	*FGFR3*	*TACC3*	122	431	FGFR3:4:1806661_TACC3:4:1732899

Supplementary Table 3.
Expression of fusion transcripts in *FGFR3-TACC3* -fusion-transfected or fusion kinase-dead (KD)-transfected cell lines, by PRADA analysis

Cell line	Pathway name	NES	FDR q-value (-$\log_2$)
Ect1/E6E7	BIOCARTA_STEM_PATHWAY	1.55	4.47
Ect1/E6E7	BIOCARTA_INFLAM_PATHWAY	1.50	4.47
Ect1/E6E7	BIOCARTA_CYTOKINE_PATHWAY	1.44	2.35
Ect1/E6E7	BIOCARTA_NTHI_PATHWAY	1.41	2.17
SiHa	BIOCARTA_INFLAM_PATHWAY	1.50	4.54
SiHa	BIOCARTA_ERYTH_PATHWAY	1.44	3.43
SiHa	BIOCARTA_SPRY_PATHWAY	1.34	2.29
ME180	BIOCARTA_ERYTH_PATHWAY	1.49	3.23
ME180	BIOCARTA_SPRY_PATHWAY	1.50	3.12
ME180	BIOCARTA_INFLAM_PATHWAY	1.46	3.05
ME180	BIOCARTA_NKT_PATHWAY	1.50	2.99
ME180	BIOCARTA_TH1TH2_PATHWAY	1.54	2.83
ME180	BIOCARTA_TNFR2_PATHWAY	1.40	2.70
ME180	BIOCARTA_NTHI_PATHWAY	1.39	2.62
ME180	BIOCARTA_LAIR_PATHWAY	1.54	2.61
ME180	BIOCARTA_CD40_PATHWAY	1.40	2.60
ME180	BIOCARTA_41BB_PATHWAY	1.56	2.59
ME180	BIOCARTA_TEL_PATHWAY	1.38	2.52
ME180	BIOCARTA_IL17_PATHWAY	1.38	2.48
ME180	BIOCARTA_STEM_PATHWAY	1.54	2.31
ME180	BIOCARTA_CYTOKINE_PATHWAY	1.57	2.20
ME180	BIOCARTA_HSP27_PATHWAY	1.32	2.19
ME180	BIOCARTA_NFKB_PATHWAY	1.33	2.15
ME180	BIOCARTA_IL12_PATHWAY	1.32	2.12
ME180	BIOCARTA_IGF1_PATHWAY	1.30	2.01

Supplementary Table 4.
Normalized enrichment score (NES) and q-value for each significantly overrepresented pathway in *FGFR3-TACC3* fusion-transfected cell lines

Single nucleotide variants of PI3K/AKT pathway in *FGFR3-TACC3* fusion-positive cervical cancer

	Fusion 1	Fusion 2	TCGA-DS-A3LQ	TCGA-WL-A834	TCGA-VS-A9UD	TCGA-ZJ-AAXT
PIK3CA	p.E545K	wild	wild	wild	NA	NA

NA ; Not available

Copy number alterations of PI3K/AKT pathway in *FGFR3-TACC3* fusion-positive cervical cancer

	Fusion 1	Fusion 2	TCGA-DS-A3LQ	TCGA-WL-A834	TCGA-VS-A9UD	TCGA-ZJ-AAXT
PIK3CA	amp	amp	neutral	neutral	neutral	neutral
AKT2	neutral	neutral	neutral	amp	neutral	amp
RICTOR	neutral	amp	neutral	amp	neutral	neutral
PIK3R1	amp	deletion	neutral	neutral	neutral	neutral
PDPK1	amp	neutral	neutral	neutral	neutral	neutral
FOXO3	amp	neutral	neutral	neutral	neutral	neutral
MLST8	amp	neutral	neutral	neutral	neutral	neutral

amp ; amplification

Supplementary Table 5.
Oncogenic alterations in the PI3K-AKT pathway in *FGFR3-TACC3* fusion-positive cancer.

	TCGA dataset (N = 306)	Japanese dataset (N = 103)
**Median Age**	46 (20-88)	46 (23-47)
**Histology**		
Squamous cell carcinoma	253 (82.7 %)	66 (64.1 %)
Adenocarcinoma	47 (15.4 %)	29 (28.2 %)
Adenosquamous carcinoma	6 (1.9 %)	6 (5.8 %)
Others	0 (0 %)	2 (1.9 %)
**Stage (FIGO)**		
I	163 (53.2 %)	75 (72.8 %)
II	70 (22.9 %)	25 (24.3 %)
III	45 (14.7 %)	1 (1.0 %)
IV	21 (6.9 %)	2 (1.9 %)
NA	7 (2.3 %)	0 (0 %)

Supplementary Table 6.
Clinicopathological characteristics of the two cervical cancer datasets used in this study

Target	Forward primer (5’ to 3’)	Reverse primer (3’ to 5’)
FGFR3-TACC3 screening<br>*FGFR3* (exon 17)-*TACC3* (exon 16)	GACCTGGACCGTGTCCTTAC	CCGTGGAGGTCAGATCTTCT
Fusion 1<br>*FGFR3* (exon 17)-*TACC3* (exon 4)	GACCTGGACCGTGTCCTTAC	AAGGGTCTGAGTGCCATCTG
Fusion 2<br>*FGFR3* (exon 17)-*TACC3* (exon 7)	GACCTGGACCGTGTCCTTAC	GGAAGTTCCAAACTGCTCCA
FGFR3 (fusion + wild-type)	GACGTGCACAACCTCGACTA	CCAAAGGACCAGACGTCACT
TACC3 (fusion + wild-type)	AGCCTGAGGAAGGAGCAGA	GAGATGAGGTCGTCGCAGAT
ACTB	TGGCACCCAGCACAATGAA	CTAAGTCATAGTCCGCCTAGAAGCA
CSF3	CCACCTACAAGCTGTGCCA	CCTGGTAGAGGAAAAGGCCG
IL1A	CTGATGATGACCTGGAGGCC	TGAGGGCGTCATTCAGGATG
IL6	GGTACATCCTCGACGGCATC	GGTTGTTTTCTGCCAGTGCC
IL8	CTCCAAACCTTTCCACCCCA	TTCCTTGGGGTCCAGACAGA
IL11	TCGAGTTTCCCCAGACCCTC	GGCAGGGAATCCAGGTTGTG
IL12A	ACTCCCAAAACCTGCTGAGG	TGGTAAACAGGCCTCCACTG
*PIK3CA (*exon 9*)*	TCATCTGTGAATCCAGAGGGG	AGCACTTACCTGTGACTCCA
FGFR3 K508M (Mutagenesis)	GCCGTGATGATGCTGAAAGACGATGCCACT	CAGCATCATCACGGCTACGGTGACAGGCTT

Supplementary Table 7.
Details of primers used in this study
